# Phenotypic and transcriptomic profiling of induced pluripotent stem cell (iPSC)-derived NK cells and their cytotoxicity against cancers

**DOI:** 10.1186/s13287-024-04029-z

**Published:** 2024-11-13

**Authors:** Nontaphat Thongsin, Siriwal Suwanpitak, Punn Augsornworawat, Jakkrapatra Srisantitham, Kritayaporn Saiprayong, Piroon Jenjaroenpun, Methichit Wattanapanitch

**Affiliations:** 1 Siriraj Center for Regenerative Medicine, Research Department, Faculty of Medicine Siriraj Hospital, Mahidol University, Bangkok, 10700 Thailand; 2 Department of Immunology, Faculty of Medicine Siriraj Hospital, Mahidol University, Bangkok, Thailand; 3 Division of Medical Bioinformatics, Research Department, Faculty of Medicine Siriraj Hospital, Mahidol University, Bangkok, Thailand

## Abstract

**Background:**

Adoptive immunotherapy using natural killer (NK) cells has attracted considerable interest in numerous clinical trials targeting both hematological and solid tumors. Traditionally, NK cells are primarily derived from either peripheral blood (PB) or umbilical cord blood (UCB). However, these methods can lead to variability and heterogeneity within the NK cell population. In contrast, induced pluripotent stem cell (iPSC)-derived NK (iNK) cells provide a more controlled and uniform cellular population, suitable for large-scale clinical applications. This makes iNK cells a promising option for developing “off-the-shelf” immunotherapeutic products. Nevertheless, current NK cell differentiation protocols, which rely on embryoid body (EB) cultures, are labor-intensive and susceptible to unwanted heterogeneity during differentiation. Here, we developed a more efficient approach for generating iNK cells by employing a monolayer and feeder-free differentiation protocol, alongside optimized culture media.

**Methods:**

The iNK cells were generated using a two-step in vitro monolayer feeder-free system following NK cell development. To evaluate their maturity, phenotypic analysis was performed using flow cytometry, comparing with PB-NK cells and the NK-92 cell line. Additionally, single-cell RNA sequencing was performed to examine their transcriptomic profiles. The cytotoxic activity of the iNK cells was evaluated by co-culturing with cholangiocarcinoma (CCA) and breast cancer (BCA) cell lines in both monolayer (2D) and tumor spheroid (3D) co-culture systems.

**Results:**

We successfully differentiated iPSCs into mesoderm (ME), hematopoietic stem/progenitor cells (HSPCs), and NK cells. The resulting iNK cells exhibited typical NK cell markers such as CD45, CD56, and CD16, and expressed key functional proteins, including both activating and inhibitory receptors. Single-cell RNA sequencing confirmed that the transcriptomic profile of our iNK cells closely resembles that of PB-NK cells. Importantly, our iNK cells demonstrated strong cytotoxic abilities against various CCA and BCA cell lines, surpassing the NK-92 cell line in both monolayer cultures and tumor spheroid cultures.

**Conclusion:**

This study highlights the potential of iPSCs as an effective alternative cell source for generating NK cells. Using a two-step in vitro monolayer feeder-free system, we successfully generated iNK cells that not only expressed key NK cell markers and their receptors but also displayed a transcriptomic profile closely resembling PB-NK cells. Furthermore, iNK cells exhibited cytotoxicity against CCA and BCA cell lines comparable to that of PB-NK cells. This approach could pave the way for off-the-shelf NK cell products, potentially enhancing the effectiveness of adoptive NK cell therapy.

**Supplementary Information:**

The online version contains supplementary material available at 10.1186/s13287-024-04029-z.

## Introduction

Natural killer (NK) cells are innate lymphoid cells that can mediate cytotoxic activity against infected cells and several types of tumor cells without HLA restriction and prior sensitization. Human NK cells can be distinguished from other lymphocytes by the expression of neural cell adhesion molecule (NCAM or CD56) and the absence of CD3 molecule (CD56^+^ CD3^−^) [1, 2]. The distinct subset of functional NK cells can be classified into two main subpopulations, CD56^bright^ and CD56^dim^. The majority of the NK cell population in peripheral blood is CD56^dim^, which mediates cytotoxic activity by secreting perforin and granzyme B, as well as other inflammatory cytokines upon activation. Meanwhile, the CD56^bright^ population is more specialized in producing cytokines [3]. Even though NK cells lack genetically rearranged receptors, the cells can recognize abnormal cells through repertoires of the germline-encoded unique set of activating and inhibitory receptors, including killer immunoglobulin receptor (KIRs), natural cytotoxicity receptors (NCRs), and the Fc gamma receptor (FcγRIIIa) CD16a. The signals received through these receptors can regulate the NK cell function [4].

Numerous clinical studies demonstrated that adoptive cell therapy using allogeneic NK cells can effectively treat acute myeloid leukemia (AML) and other malignancies without causing graft-versus-host disease (GvHD) [5, 6]. Currently, the common sources of NK cells are primary NK cells isolated from peripheral blood (PB-NK cells), umbilical cord blood (UCB-NK cells), and NK-92 cell line. While the NK cells from each source exhibit effective clinical outcomes, certain limitations remain. PB-NK cells have been used primarily in clinical trials; however, their application is constrained by the limited cell yield and donor variability [7]. UCB-NK cells have limited anti-tumor activity due to their immature phenotype and elevated expression of the inhibitory receptor NKG2A [8, 9]. Furthermore, the use of the immortalized NK-92 cell line, derived from a patient with non-Hodgkin's lymphoma, requires sub-lethal irradiation before infusion to prevent in vivo proliferation [10]. Therefore, an alternative NK cell source is required to overcome these limitations. Pluripotent stem cells, including embryonic stem cells (ESCs) and induced pluripotent stem cells (iPSCs), have unlimited proliferation capacity and can be differentiated into all cell types in the body. Recently, functional NK cells derived from human ESCs and iPSCs have been generated using feeder cells such as murine stromal cells **(**M210-B4, EL08-1D2, and OP9 cells) [11, 12] or feeder-free systems such as spin-EB [13]. ESC- and iPSC-derived NK cells exhibit similar phenotypic profiles, such as expression of KIRs, CD16, NKp44, NKp46, NKG2D, FasL, and TRAIL. These cells also demonstrate comparable effector function, including the ability to eliminate various types of tumors in vitro and in vivo compared to PB-NK cells [14–16]. The iPSC technology also provides the ability to produce NK cells on a large scale, allowing for preparation in advance and reducing batch-to-batch variations. Moreover, iPSCs can be manipulated using genome editing technology to augment NK cell effector function and persistence. Previous studies have shown that the expression of chimeric antigen receptor (CAR) improves NK cell-killing activity against various tumor cells in both in vitro and in vivo settings. Additionally, stable expression of CD16 (non-cleavable CD16) in iNK cells enhances antibody-dependent cell-mediated cytotoxicity (ADCC). Moreover, knocking out the cytokine-inducible SH2-containing protein (*CISH*) gene leads to improved iNK cell proliferation and anti-tumor activity [17–19]. These platforms offer more effective “off-the-shelf” cell products for cancer immunotherapy.

To date, spin-EBs have been used to generate iNK cells for clinical studies. However, the differentiation outcomes are highly dependent on the quality of the EBs, such as their size and heterogeneity, which could hamper the success rate [20–22]. Here, we establish a simplified monolayer feeder-free system to induce the differentiation of hiPSCs to hematopoietic stem/progenitor cells (HSPCs), which could be further developed into iNK cells. The generated iNK cells exhibited typical NK cell characteristics, including the expression of developmental markers, inhibitory receptors, and activating receptors. Notably, these iNK cells displayed potent cytotoxic activity against cholangiocarcinoma and breast cancer cell lines. Transcriptomic analysis revealed heterogeneity within the iNK cell population, highlighting similarities between mature and adaptive cell populations found in iNK cells and PB-NK cells.

## Methods

### Cell culture

iPSCs generated from cesarean scar fibroblasts (MUSIi001-A) [23] were maintained on Matrigel® matrix (1:40 dilution, Corning)-coated plate in Essential 8™ medium (Gibco™). iPSCs were subcultured every 3—4 days using 0.5 mM EDTA (Gibco™). NK-92 cell line was purchased from American Type Tissue Culture (ATCC, CRL-2408). The cells were maintained in a complete α-MEM medium containing α-MEM (Gibco™), 10% fetal bovine serum (FBS) (Gibco™), 10% horse serum (Sigma Aldrich), 2 mM GlutaMAX™ (Gibco™), and 1% penicillin/streptomycin (Gibco™), supplemented with 100 U/ml hIL-2 (PeproTech). The cells were subcultured every 4 days. Cholangiocarcinoma (CCA) cell lines, KKU-055 (JCRB1551), KKU-100 (JCRB1568), KKU-213A (JCRB1557), were obtained from the Japanese Collection of Research Bioresources (JCRB) Cell Bank (Osaka, Japan). They were maintained in a complete DMEM/F12 medium containing DMEM/F12 (Gibco™), 10% FBS, 2 mM GlutaMAX™ (Gibco™), and 1% penicillin/streptomycin (Gibco™). The CCA cell lines were subcultured every 4 days using 0.1% trypsin/EDTA (Gibco™). Breast cancer cell lines, MCF-7 (#HTB-22) and MDA-MB-231 (#HTB-26) were purchased from ATCC. They were maintained in DMEM (Gibco™), 10% FBS (Gibco™), and 1% penicillin/streptomycin (Gibco™).

### Isolation of PBMCs

The use of peripheral blood samples was approved by the Siriraj Institutional Review Board (SiRB), Faculty of Medicine Siriraj Hospital, Mahidol University, Thailand (Si 320/2021), in accordance with the Helsinki Declaration of 1975. Written informed consent was obtained from healthy donors. Peripheral blood NK (PB-NK) cells were isolated from peripheral blood mononuclear cells (PBMCs) using the MojoSort™ Human NK Cell Isolation Kit (BioLegend). PB-NK cells were maintained in a complete RPMI-1640 medium containing RPMI-1640 (Gibco™), 10% FBS, 2 mM GlutaMAX™, 1% non-essential amino acid (Gibco™), and 1% penicillin/streptomycin, supplemented with 100 U/ml hIL-2. NK cells were expanded by co-culturing with irradiated artificial antigen-presenting cells expressing membrane-bound IL-21 (K562-mIL-21; aAPCs) at 1:2 Effector: Target (E:T) ratio in a complete RPMI medium supplemented with 100 U/ml hIL-2 for 6 days. Afterward, aAPCs were added to the culture at 1:1 E:T ratio and cultured for an additional 6 days.

### Generation of iPSC-derived NK cells (iNK) using a monolayer feeder-free system

iPSCs were pre-treated with 10 μM Y-27632 (Abcam) for 1 h and dissociated into single cells using TrypLE™ Select (Gibco™). The cells were seeded onto a Matrigel® matrix (1:40 dilution)-coated plate at a density of 4.2 × 10^4^ cells/cm^2^ in Essential 8™ medium supplemented with 10 μM Y-27632. After 24 h, Y-27632 was withdrawn, and the cells were cultured in Essential 8™ medium for an additional 2 days. On day 0, iPSCs were induced toward hematoendothelial progenitor (HEP) cells based on our previous publication [24]. In brief, iPSCs were cultured in RPMI-1640, 2% B-27 (Gibco™), 2 mM GlutaMAX™ (Gibco™), and 60 μg/ml ascorbic acid (Gibco™) supplemented with 6 μM CHIR99021 (Sigma Aldrich) for two days. On day 2, the cells were cultured in the same medium supplemented with 50 ng/ml VEGF (BioLegend) and 10 ng/ml FGF2 (BioLegend). On day 3, the cells were dissociated into single cells and seeded onto a Matrigel® matrix (1:40 dilution)-coated culture plate at a density of 4 × 10^4^ cells/cm^2^. On day 5, the medium was changed to culture medium supplemented with 50 ng/ml VEGF, 10 ng/ml FGF2, and 10 µM TGF-β inhibitor (SB431542) (Merck Millipore), and cultured for an additional 7 days. The culture medium was changed every 2 days. On day 12, the culture medium was changed to DMEM (Gibco™) containing HAM’s F-12 (Gibco™), 15% heat-inactivated human AB serum (Gibco™),  1% penicillin/streptomycin (Gibco™), 2 mM GlutaMAX™ (Gibco™), 1 mM β-mercaptoethanol, 5 ng/ml sodium selenite, 50 μM ethanolamine, and 20 µg/ml ascorbic acid supplemented with 5 ng/ml hIL-3, 10 ng/ml hIL-15, 10 ng/ml hFlt3L, 20 ng/ml hSCF, and 20 ng/ml hIL-7 (all from BioLegend) and cultured for 7 days. Afterward, hIL-3 was withdrawn, and the cells were cultured in the same culture medium for an additional 3 weeks as previously published work [13]. Floating cells were collected every 7 days for flow cytometric analysis. On day 40, the floating cells were collected for transcriptomic analysis. The cytotoxic activity against CCA and BCA cell lines was determined.

### Reverse transcription-quantitative polymerase chain reaction (RT-qPCR)

Total RNA was extracted using the RNeasy Mini Kit (Qiagen). Complementary DNA (cDNA) was synthesized using the RevertAid First Strand cDNA Synthesis Kit (Thermo Fisher Scientific) according to the manufacturer’s instructions. qPCR was performed using the KAPA™ SYBR® FAST qPCR Master Mix Kit (Sigma Aldrich) on the CFX96™ Real-Time PCR detection system (Bio-Rad). The cycle parameters were an activation step at 95 °C for 1 min, followed by 40 cycles of a denaturation step at 95 °C for 2 s, an annealing step at 60 °C for 30 s, and an extension step at 70 °C for 2 s. Data were normalized with the housekeeping gene, *GAPDH,* and the expression levels were compared to those of the undifferentiated iPSCs. Primer sequences are listed in the Additional file 1: Table. S1.

### Flow cytometry

Cells were harvested and resuspended in fluorescent activated cell sorting (FACS) buffer (3% FBS in 1XPBS) containing 10% human AB serum (Sigma Aldrich), and incubated at 4 °C for 30 min to block the non-specific binding. Afterward, the cells were stained with fluorescence-conjugated and isotype-matched antibodies with the same fluorescence dye. Dead cells were stained with the Zombie Fixable Viability Kit (BioLegend), and incubated at room temperature for 15 min in the dark. After staining, cells were washed with FACS buffer and fixed with 1% paraformaldehyde. Flow cytometric analysis was performed using the BD LSRFortessa™, and the data were analyzed using FlowJo software. Details of all antibodies are listed in Additional file 1: Table S2.

### Single-cell RNA (sc-RNA) sequencing and data analysis

iNK cells and PB-NK cells were collected, washed, and resuspended in 0.04% bovine serum albumin (BSA) in 1XPBS at a cell density of 1 × 10^6^ cells/ml. The cells were then processed for cell barcoding, loaded to a 10X chromium machine (10X genomics), and run through library preparation according to the manufacturer’s instructions of the Chromium Next GEM Single Cell 5’ Reagent Kits v2. Afterward, the library was sequenced using next-generation sequencing (NGS).

Following sequencing, the raw data files were processed and analyzed using the 10X Cell Ranger. The processed dataset files were analyzed using Rstudio (version 2023.09.) running R (version 4.3.0). Single-cell RNA sequencing analysis was performed using Seurat (v 4.3.0.1) and Monocle 3 (v 1.3.1). Briefly, processed dataset files were imported using ‘Read10x’ and compiled to generate a Seurat object file using ‘CreateSeurateObject’. Poor quality cells, including stressed, apoptotic, or dead cells and doublets, were excluded by removing cells with high mitochondrial RNA content (> 25%) and abnormal RNA counts (nCount_RNA > 20,000 and nFeature_RNA > 6000 and < 1000 for both conditions), respectively. Gene expression data were scaled and normalized using ‘SCTransform’, clusters were computed using ‘RunPCA’, ‘RunUMAP’ (dims = 1:30 and 1:40 for iNK and primary NK, respectively), ‘FindNeighbors’, and ‘FindClusters’ (resolution = 0.2 and 0.6 for iNK and primary NK, respectively). Clusters were identified by validating the gene expression of generic markers for each immune cell type. Moreover, ambiguous or unidentified populations were classified by observing differentially expressed genes using ‘FindMarkers’.

For pseudotime analysis, the Monocle3 package was used to reconstruct the NK population and perform pseudotime ordering. Briefly, the iNK population was isolated using ‘subset’ converted to a monocle3 compatible CellDataSet object using ‘as.cell_data_set’ of the Seurat-wrapper package. Cell reconstruction and pseudotime ordering were performed using ‘cluster_cells’ (using the Louvain cluster method), and ‘learn_graph’, respectively. The initial pseudotime point was assigned using ‘order_cells’. The pseudotime point information was then transferred to the Seurat object as metadata using ‘AddMetaData’ for further comparative analysis.

To integrate single-cell RNA sequencing datasets for comparative analysis, datasets of iNK and primary NK conditions were compiled using ‘SelectIntegrationFeatures’, ‘PrepSCTIntegration’, ‘FindIntegrationAnchors’, and ‘IntegrateData’. The SCT method was used for data normalization. Cellular populations were identified and analyzed using gene expression of generic and identified markers. Comparative analysis was performed using ‘FindMarkers’ to determine differentially expressed genes and the ‘EnhancedVolcano’ package (version 1.22) to generate volcano plots.

### Cytotoxicity assay

The killing assay was performed against CCA cell lines (KKU-055, KKU-100, KKU-213A) and BCA cell lines (MCF-7 and MDA-MB-231) in 2D and 3D tumor models. For the 2D tumor model, 7 × 10^3^ cancer cells were cultured in a flat-bottom 96-well plate overnight. The next day, the iNK cells, PB-NK cells, and NK-92 cell line were co-cultured with CCA cell lines at 1:1, 2.5:1, 5:1, and 10:1 E:T ratios and BCA cell lines at 1:1, 2:1, 5:1, and 10:1 E:T ratios for 6 h. Afterward, the cells were washed with 1XPBS and stained with crystal violet at room temperature for 15 min in the dark. The stained cells were washed and dried at 37 °C for 30 min. Crystal violet was dissolved with absolute methanol, and the absorbance was measured using a microplate reader (Synergy™ H1, Biotek) at 570 nm. Killing efficiency was calculated using the formula: % specific killing = ((O.D of control – O.D of the sample)/O.D of control) × 100.

For the cytotoxic activity of NK cells against tumor spheroids (3D tumor model), the KKU-213A and MDA-MB-231 cell lines were dissociated into single cells. The cells were labeled with 2.5 μM carboxyfluorescein succinimidyl ester (CFSE) according to the manufacturer’s instructions. For spheroid formation, 2 × 10^5^ CFSE-labeled KKU-213A and MDA-MB-231 cells were resuspended with 10 ml of an ice-cold complete DMEM/F12 containing 2.5% Matrigel® matrix. The CFSE-labeled KKU213-A and MDA-MB-231 cells were seeded at a density of 2 × 10^3^ cells/well of an ultra-low attachment round-bottom 96-well plate, and centrifuged at 1,000 × g at 4 °C for 10 min. The spheroids were cultured for 2 days before the killing assay. NK cells were co-cultured with the CFSE-labeled KKU-213A spheroids at a 1:1, 2.5:1, and 5:1 E:T ratios, and with CFSE-labeled MDA-MB-231 spheroids at a 1:1, 2:1, and 5:1 E: T ratios in the RPMI-1640 (Gibco™), 10% FBS, 2 mM GlutaMAX™, 1% non-essential amino acid (Gibco™), and 1% penicillin/streptomycin supplemented with 1 μg/ml propidium iodide (PI) for 3 days. Afterward, images were acquired using a confocal microscope (Nikon Instruments Inc., Melville, NY, USA). The mean fluorescence intensity (MFI) of PI was quantified using the NIS-Elements software. Killing efficiency was calculated using the formula: % specific killing = ((Experimental MFI − Spontaneous MFI)/(Maximum MFI − Spontaneous MFI)) × 100, where experimental MFI was obtained from the co-culture condition and spontaneous MFI was obtained from the tumor spheroids alone.

### Enzyme-linked immunosorbent assay (ELISA)

Cytokine secretion by NK cells was examined using ELISA. The culture supernatant was collected at the endpoint of the cytotoxicity assay against CCA cell lines (KKU-055, KKU-100, and KKU-213A) at 5:1 E:T ratio and BCA cell lines (MCF-7 and MDA-MB-231) at 10:1 E:T ratio. The secretion of IFN-γ or TNF-α by NK cells was measured by DuoSet® Development Systems kits (R&D Systems) following the manufacturer’s instructions. The results were analyzed using a microplate reader (Synergy™ H1, Biotek).

### Statistical analysis

Statistical analyses were performed using the GraphPad Prism software (v8.0). The data are shown as the mean ± SD or mean ± SEM. The one-way ANOVA or two-way ANOVA with Tukey’s correction for multiple comparisons was used for the comparison between the two groups. For multiple comparisons, p-values are indicated in the corresponding figures.

## Results

### Differentiation of iPSCs toward HSPCs using a feeder-free culture system

**Fig. 1:**
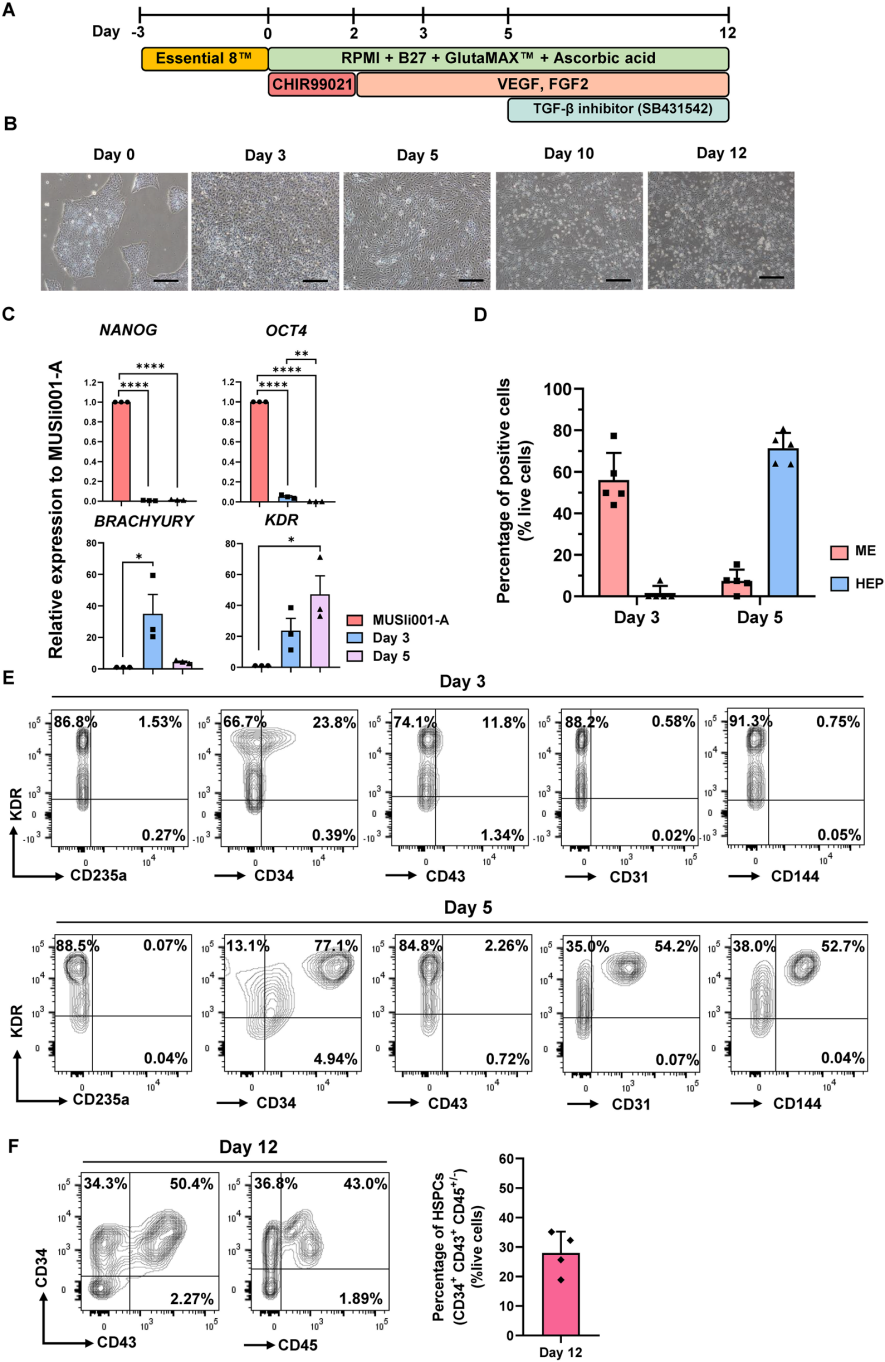
In vitro differentiation of iPSCs toward HSPCs. (**A**) Schematic diagram of a feeder-free protocol for generating iPSC-derived HSPCs. (**B**) Representative images show the differentiation of iPSCs toward HSPCs: day 0 (iPSCs), day 3 (mesoderm, ME), day 5 (hemogenic endothelial progenitor cells, HEPs), day 10, and day 12 (hematopoietic stem/progenitor cells, HSPCs). Scale bar = 200 μm. (**C**) qPCR analysis of mRNA expression of pluripotent genes (*NANOG* and *OCT4*), mesendodermal gene (*BRACHYURY*), and mesodermal gene (*KDR*) on days 0, 3, and 5 of differentiation, mean ± SEM (n = 3 independent experiments). (**D**) Flow cytometric analysis shows the percentage of ME (KDR^+^ CD235a^−^ CD34^−^) and HEPs (KDR^+^ CD235a^−^ CD34^+^ CD31^+^ CD144^+^) on days 3 and 5 of differentiation (n = 5 independent experiments). Boolean gating was performed using the FlowJo software to obtain the percentage of ME and HEP. (**E**) Representative flow cytometric analysis of ME and HEPs on days 3 and 5 of differentiation. The plots are representative of one experiment within n = 5 independent experiments (**F**) Flow cytometric analysis shows the percentage of HSPCs (CD34^+^ CD43^+^ CD45^+/-^) on day 12 of differentiation (n = 4 independent experiments). The plots are representative of one experiment within n = 4 independent experiments). The positive population of flow cytometry data was gated using the isotype control. Boolean gating was performed using the FlowJo software to obtain the percentage of HSPCs. For D and F, graphs represent the mean ± SD. One-way ANOVA (Tukey’s multiple comparison test) was performed: ****p < 0.0001, ** p < 0.01, * p < *0.05*

For directed differentiation of iPSCs to NK cells, a two-step in vitro monolayer feeder-free system was performed. In the initial step, iPSCs were differentiated toward mesoderm (ME) by activating the Wnt signaling pathway using the GSK3 inhibitor (CHIR99021), followed by the induction toward hemogenic endothelial progenitor (HEP) cells and hematopoietic stem/progenitor cells (HSPCs) using VEGF, FGF2, and TGF-β inhibitor (SB431542) (Fig. 1A).

Within the first 5 days of ME and HEP induction, the morphology of iPSCs dynamically changed to mesodermal-like cells and endothelial-like cells, which could be observed on days 3 and 5, respectively (Fig. 1B). Additionally, the expression of pluripotent genes (*NANOG* and *OCT4*) was drastically down-regulated throughout the differentiation process (Fig. 1C). By day 3 of differentiation, 56.0 ± 13.1% of cells expressed ME markers (KDR^+^ CD235a^−^ CD34^−^) (Fig. 1D, E and Additional file 2: Fig. S1A). By day 5, re-seeding of mesodermal cells greatly promoted the differentiation of ME toward HEPs (KDR^+^ CD235a^−^ CD34^+^ CD31^+^ CD144^+^), resulting in an approximately 71.2 ± 7.3% HEPs, while the percentage of ME sharply decreased to 1.5 ± 3.4% (Fig. 1D, E and Additional file 2: Fig. S1A). In addition, the expression of a primitive streak gene, *BRACHYURY*, was significantly upregulated on day 3 following Wnt signaling activation, and *KDR* expression was significantly increased over time (Fig. 1C). Taken together, our data indicated that iPSCs were committed to HEPs through in vitro mesodermal patterning using CHIR99021, VEGF, and FGF2.

The HEPs were continuously cultured in the presence of TGF-β inhibitor (SB431542) for an additional 7 days to promote the differentiation toward HSPCs through endothelial-to-hematopoietic transition (EHT). We observed the emergence of floating cells from day 10 onward (Fig. 1B). On day 12, these floating cells were then collected for phenotypic analysis by flow cytometry. The results revealed the emergence of the HSPC population (CD34^+^ CD43^+^ CD45^+/-^) in the culture, accounting for 27.99 ± 7.2% of the whole population, while the non-floating cells on days 8 and 12 exhibited similar phenotypic profiles to HEP on day 5 (Fig. 1F and Additional file 2: Fig. S1B and S2).

### Monolayer feeder-free system promotes the differentiation of HSPCs toward NK cells

**Fig. 2:**
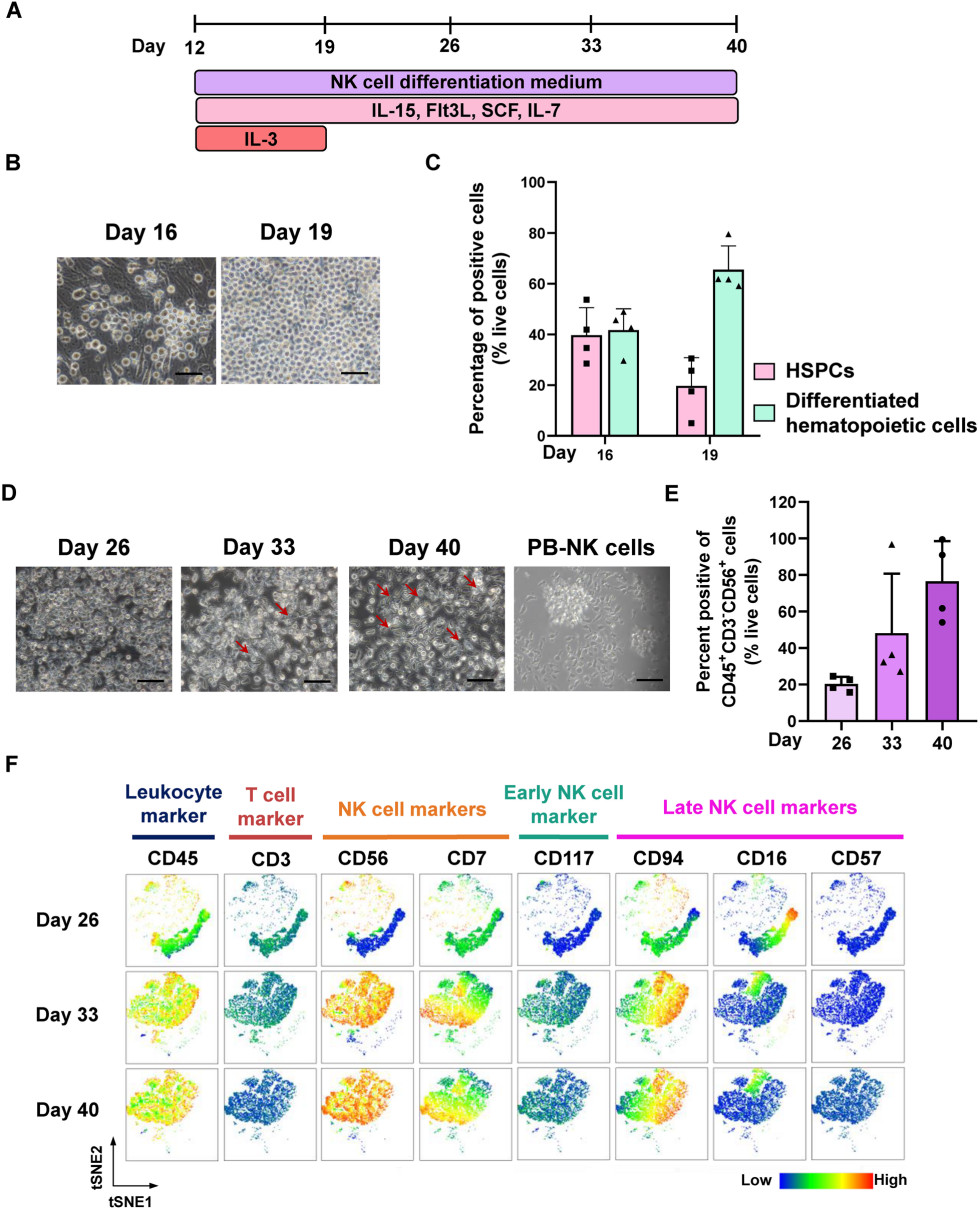
In vitro differentiation of HSPCs toward NK cells. (**A**) Schematic diagram of a feeder-free protocol for generating HSPC-derived NK cells. (**B**) Representative images show the emergence of floating cells from the adherent cells on days 16 and 19 of differentiation. Scale bar = 50 μm. (**C**) The percentage of HSPCs (CD34^+^ CD43^+^ CD45^+/-^)and differentiated hematopoietic cells (CD34^−^ CD43^+^ CD45^+^) on days 16 and 19 (n = 4 independent experiments). Boolean gating was performed using the FlowJo software to obtain the percentage of HSPCs and differentiated hematopoietic cells. (**D**) Representative images show the morphological change of floating cells from round-shaped to NK-like morphology (the red arrow indicates the NK-like morphology). (**E**) The percentage of iNK cells (CD45^+^ CD3^−^ CD56^+^) on days 26, 33, and 40 (n = 4 independent experiments). Boolean gating was performed using the FlowJo software to obtain the percentage of NK cells. (**F**) t-SNE analysis revealed two distinct cell clusters expressing NK cell developmental markers throughout differentiation. All data represent the mean ± SD

We induced the differentiation of HSPCs toward NK cells by switching to an NK cell differentiation medium supplemented with cytokines for 4 weeks (Fig. 2A) [13]. During the differentiation, floating cells began to emerge from adherent cells between days 16–19 (Fig. 2B). Flow cytometric analysis on day 16 revealed two main populations: approximately 40% HSPCs (CD34^+^ CD43^+^ CD45^+/-^) and 40% differentiated hematopoietic cells (CD34^−^ CD43^+^ CD45^+^). After an additional 3 days of culture, the HSPC population decreased to 19.68 ± 11.15%, while the differentiated hematopoietic cell population increased to approximately 65.52 ± 9.40% (Fig. 2C). These results suggest that the HSPCs could undergo proliferation and differentiation toward differentiated hematopoietic cells, providing a starting cell source for generating other types of immune cells.

After one week of differentiation, IL-3 was removed, and the differentiation medium was changed every 3 days for an additional 3 weeks. From day 33 onward, we observed morphological changes from round-shaped (day 26) to NK-like morphology (day 33 and 40), similar to PB-NK cells (Fig. 2D). Correspondingly, the NK cell population (CD45^+^ CD3^−^ CD56^+^) significantly increased throughout differentiation (Fig. 2E). A high dimensional flow cytometric analysis using t-stochastic neighbor embedding (tSNE) revealed two major cell clusters across differentiation time points. On day 26, the largest cell cluster exhibited low CD56 expression, indicative of a non-NK cell population. After an additional 7 days of differentiation, this cell cluster highly expressed CD45 and CD56, indicating the transition to NK (iNK) cells; the expression of CD45 and CD56 persisted throughout the differentiation. Within the iNK cluster, we observed a gradual increase in the expressions of CD7, CD94, and CD16, ranging from low to high levels. Moreover, iNK cells exhibited low levels of CD117 (early NK cell marker) and CD3 (T cell marker). These results suggested a spectrum of cellular states within the iNK cell population (Fig. 2F). The percentages of CD117^+^ cells in the CD56^+^ population ranged from 12.4% to 41.8%, reflecting the variation in each differentiation batch (Additional file 2: Fig. S3A). By day 40, it was also determined that a single iPSC could generate 37 iNK cells using the monolayer feeder-free protocol (Additional file 2: Fig. S3B).

### Phenotypic analysis of NK cell markers

**Fig. 3:**
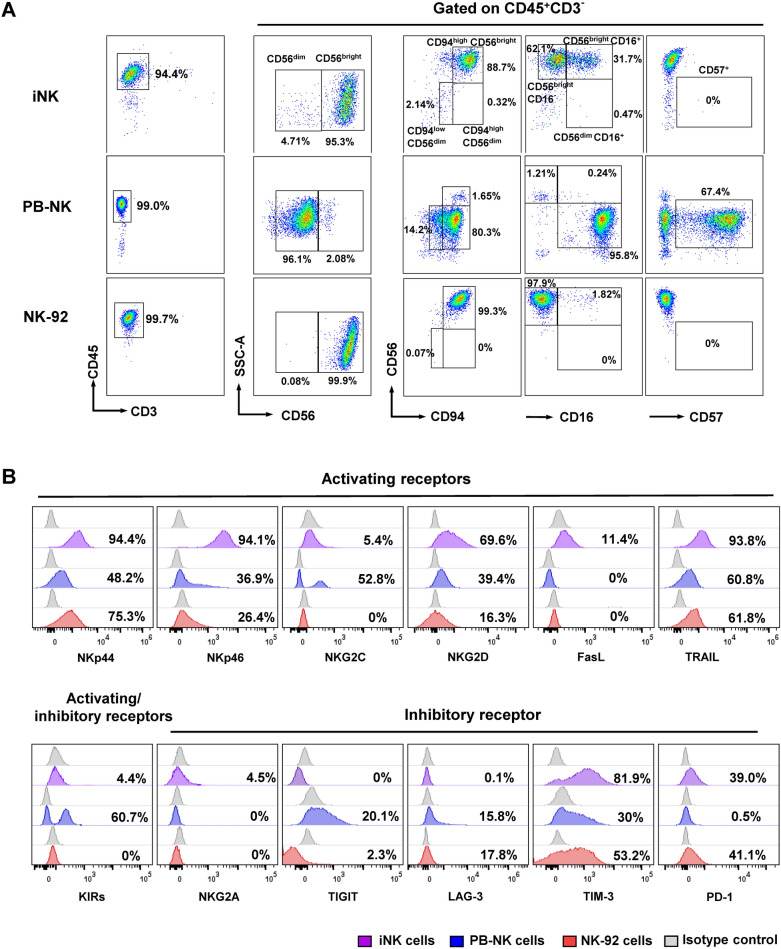
Phenotypic analysis of NK cells. (**A**) Representative flow cytometric analysis shows the phenotype of the iNK cells on day 40 of differentiation, PB-NK cells, and the NK-92 cell line, gated on the CD45^+^ CD3^−^ population. (**B**) Representative flow cytometric analysis shows the expression of activating and inhibitory receptors of the iNK cells on day 40 of differentiation, PB-NK cells, and NK-92 cell line. The positive population of flow cytometric data was gated using the isotype control. The plots are representative of one experiment within n = 2 independent experiments. The anti-KIRs monoclonal antibody is an antibody cocktail targeting an inhibitory receptor KIR2DL1, and the activating receptors KIR2DS2, KIR2DS3, and KIR2DS5

Next, we analyzed the phenotype of iNK cells on day 40, in comparison to the PB-NK cells and the NK-92 cell line. CD56 expression levels have been used to identify the developmental stages of NK cells: the CD56^bright^ subset is immature, while the CD56^dim^ subset is more mature [25]. We first gated CD45^+^ CD3^−^ cells to assess the NK cell population. We observed elevated CD56 expression levels (CD56^bright^) in the iNK cells and the NK-92 cell line, constituting 95.3% and 99.9%, respectively, suggesting that they are relatively immature. In contrast, the PB-NK cells exhibited 96.1% CD56^dim^ cells (Fig. 3A and Additional file 2: Fig. S4, S5A—C).

The acquisition of surface protein expression of CD56 and CD94 molecules has been reported to be involved in the development of NK cells in secondary lymphoid tissues (SLT) [26]. We assessed the expression levels of CD56 and CD94 in the iNK cells, PB-NK cells, and the NK-92 cell line. Flow cytometric data showed that the major population of iNK cells and NK-92 cells were CD94^high^ CD56^bright^, accounting for 88.7% and 99.3%, respectively. On the other hand, the CD94^high ^CD56^bright^ population in the PB-NK cells was 1.65%, while the CD94^high^ CD56^dim^ and CD94^low^ CD56^dim^ populations were 80.3% and 14.2%, respectively (Fig. 3A).

NK cells can initiate tumor cytotoxicity through specific Fc receptors expressed on their cell surface, such as CD16 (FcγRIIIA), which binds to the Fc portion of immunoglobulin (IgG) attached to the target antigen on cancer cells. This binding, so-called antibody-dependent cell-mediated cytotoxicity (ADCC), transmits activating signals within the NK cells, resulting in target cell lysis. The CD16 receptor is primarily found on CD56^dim^ NK cells (CD56^dim^ CD16^+^), constituting more than 90% of the NK cell population in the bloodstream [27]. Conversely, the CD56^bright^ NK cells are CD16^low^ or CD16^−^. We found that 62.1% of the iNK cells were CD56^bright^ CD16^−^, while 31.7% were CD56^bright^ CD16^+^. Meanwhile, the CD56^dim^ CD16^+^ population of the PB-NK cells constituted 95.8% (Fig. 3A). We further investigated the level of CD57 on the NK cells, a marker commonly used to determine the terminal maturation of NK cells. The expression of CD57 is associated with reduced cytotoxic activity [28, 29]. We found that the iNK cells and the NK-92 cell line did not express the CD57 molecule, while 67.4% of the PB-NK cells were CD56^+^ CD57^+^ (Fig. 3A).

Typically, NK cells express various transmembrane receptors, including activating and inhibitory receptors. The cytotoxic function of NK cells is tightly regulated by the balance between activating and inhibitory signals through these receptors [30]. We then examined the expression profile of these receptors among different NK sources. The iNK cells expressed a significantly high level of the activating receptors, NKp44, NKp46, and NKG2D, at approximately 94.4%, 94.1%, and 69.6% respectively, compared to the PB-NK cells and NK-92 cell line. The expressions of dead ligands, including FasL and TRAIL, were also detected at higher levels in comparison to PB-NK cells and NK-92 cell line, approximately 11.4% and 93.8%, respectively. The expression level of other receptors, such as NKG2C, was lower than those of the PB-NK cells. Interestingly, the inhibitory receptors on the iNK cells, TIM-3 and PD-1, were expressed at higher levels compared to PB-NK cells. We also found a low level of NKG2A, which accounted for 4.5% of the iNK cells. Nevertheless, the expressions of TIGIT and LAG-3 were lower than PB-NK cells (Fig. 3B). Additionally, there were variations in the expression of both activating and inhibitory receptors across different batches of iNK cell differentiation and among different donors (Additional file 2: Fig. S6A, B). Taken together, our results indicate that we successfully generated the iNK cells with an immature phenotype, closely resembling the NK-92 cell line, based on high expression of CD56^bright^. In contrast to the NK-92 cell line, the iNK cells expressed CD16, enabling them to mediate cytotoxicity through ADCC. In addition, they exhibited elevated levels of activating receptors, such as NKp44, NKp46, NKG2D, FasL, and TRAIL, suggesting their potential to exert cytotoxic activity against target cancer cells.

### Transcriptomic profiles of iNK cells

**Fig. 4:**
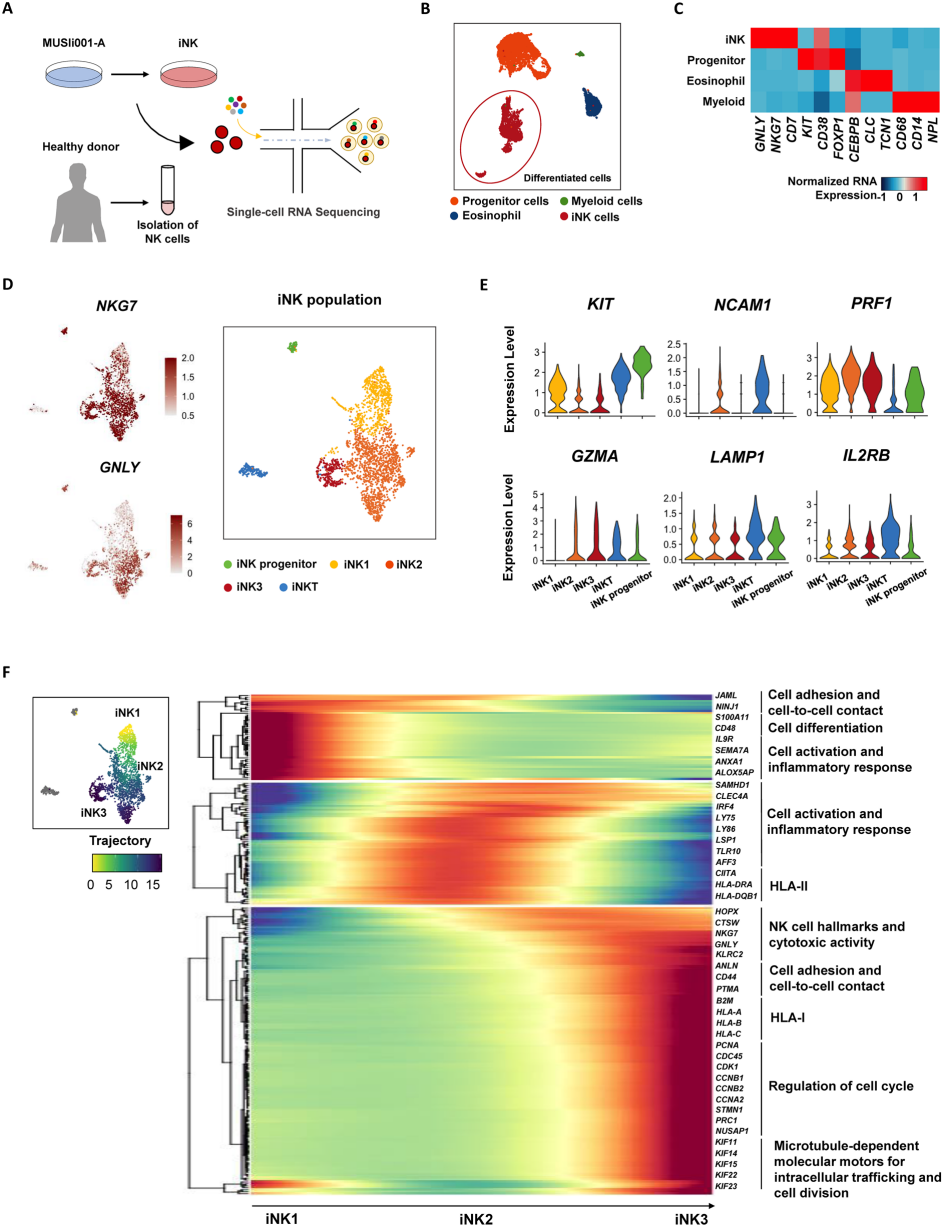
Transcriptomic analysis of iNK cells. (**A**) Schematic of single cell-RNA sequencing. (**B**) UMAPs showing identified cell types in the iNK cell culture. (**C**) Heat map showing gene expression of markers associated with each cell type. (**D**) UMAPs showing the identified cell types in the iNK cell population and the expression of *NKG7* and *GNLY* genes. (**E**) Violin plots represent gene expression associated with NK cells. (**F**) Trajectory analysis of the iNK cell clusters

To better understand the cell types present within the iNK cells, we performed single-cell RNA sequencing (Fig. 4A) and analyzed the transcriptional profiles of cells produced from our directed differentiation protocol of iPSCs into iNK (n = 8,199 cells). These differentiated samples were processed for library preparation on day 40 of the protocol, which gave rise to four major distinct populations (Fig. 4B), including NK cells (annotated as iNK). The iNK population, accounting for 29% of the total population, displays generic NK cell markers such as *GNLY*, *NKG7* and *CD7* (Fig. 4C). Other identified populations include progenitor cells (*KIT, CD38, FOXP1*), eosinophils (*CEBPB, CLC, TCN1*), and myeloid cells (*CD68, CD14, NPL*) (Fig. 4B, C). Of note, progenitor cells constitute the majority (55%) of the total population, while myeloid cells and eosinophils account for a minority of the total composition.

NK cells are diverse, encompassing a wide range of subsets, each marked by distinct markers and characterized by their unique roles in the immune system [31]. To explore this heterogeneity, we focused on the iNK population and performed a subclustering analysis to identify specific subsets of iNK cells arising from our differentiation protocol. We identified a total of five iNK subsets, each characterized by their generic markers indicating their maturation and activating states. Due to the low expression of the common activation marker *NCAM1* (CD56) gene [31] in the iNK cells, we were unable to use this marker to delineate the subsets. We classified the iNK population into three distinct states (iNK1, iNK2, and iNK3) based on the presence of other well-known NK activating markers such as *NKG7* and *GNLY* (Fig. 4D). The classified iNK subsets differ in the expression levels of genes associated with NK cell characteristics, as well as genes related to development under the hematopoietic lineage. The NK progenitor marker *KIT* (CD117) is highly expressed in iNK1 and decreases in iNK2 and iNK3 (Fig. 4E), marking the changing state in NK maturation. For other cell populations, we distinguished NKT cells and NK progenitors using their cell identity markers. Specifically, we identified NKT cells by their characteristic markers (Additional file 2: Fig. S7) and NK progenitors by the lack of markers typical for fully differentiated NK cells [32–34].

The iNK subsets form clusters that are congruent with one another, indicating a continuum of genetic transitions from one state to another. To further explore these transitions, we conducted a pseudotime ordering analysis of these populations. This approach allowed us to highlight specific genes or gene categories that serve as markers for the changes occurring through these transitional states (Fig. 4F). Throughout the trajectory, we pinpointed distinct gene clusters associated with each transitioning subset. For instance, cells within the iNK1 subset prominently express genes linked to progenitor stages, whereas iNK2 cells are characterized by gene signatures indicative of NK cell activation and inflammatory responses, including HLA genes. Meanwhile, iNK3 cells are distinguished by their expression of genes essential for NK cell cytotoxic functions, together with gene expressions associated with cell adhesion, HLA activation, regulation of the cell cycle, and microtubule-dependent molecular motors. Collectively, our comprehensive analysis explores the dynamic transitioning of cellular states found in the iNK cell differentiation, highlighting distinct functional features within the NK cell lineage and providing valuable insights into the heterogeneity and maturation of NK cells.

### iNK cells exhibit a similarity of transcriptomic profiles to PB-NK cells

Primary NK cells from peripheral blood have been extensively characterized [31, 35, 36], establishing a benchmark that guides the further analysis of our in vitro iNK cells. We obtained peripheral blood from a healthy donor, performed NK cell enrichment using magnetic beads, and sequenced primary NK cells for comparison (n = 8,424), aiming to identify discrepancies between the two sources of NK population. Consistent with the previous report [31], we identified key NK cell types and their subsets using our dataset. These subsets possess distinct identity features reflected by their maturation and activation states.

**Fig. 5:**
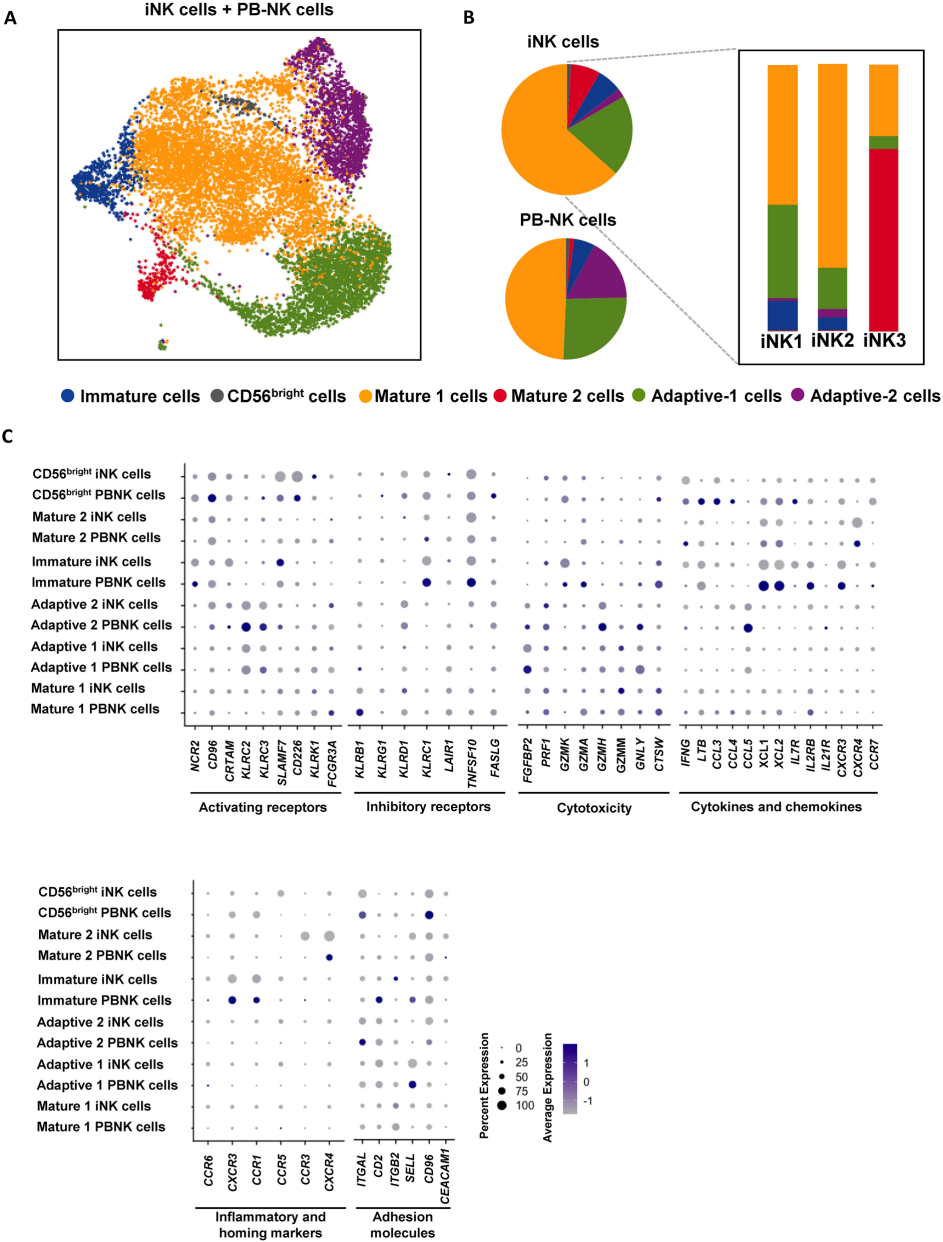
Transcriptomic analysis of iNK cells and PB-NK cells. (**A**) UMAP projections of merged iNK cells and PB-NK cells. The different colors represent the 6 NK cell subpopulations. (**B**) Cellular compositions of the iNK cells and PB-NK cells. (**C**) Dot plot showing the markers of each NK cell subpopulation. Dot size corresponds to the proportion of cells within the group expressing each gene, and dot color corresponds to expression level

To refine our understanding of the identities within our in vitro iNK populations, we merged the dataset from the iNK cells with that of primary NK cells (Fig. 5A). Using classifications from our primary NK cell analysis, we observed that certain iNK subsets aligned and formed clusters with primary NK cell subsets, highlighting their similarity at a transcriptomic level. While the cellular compositions of NK cells differ between the iNK cells and primary NK cells, both sources contain significant amounts of Mature 1 and Adaptive 1 subsets (Fig. 5B). In mapping the earlier iNK classifications (iNK1, iNK2, and iNK3) to the integrated labels, we found that a considerable portion of the iNK1 subset corresponds with the primary Adaptive 1 subset. Meanwhile, the iNK2 subset predominantly corresponds with the Mature 1 subset, and the iNK3 subset aligns most closely with the Mature 2 subset. This suggests that our in vitro iNK cells, despite originating from a stem cell differentiation process, exhibit transcriptional profiles and subsets that are closely analogous to their native counterparts.

To explore the differences in the functional features between primary NK cells and the iNK cells, we graphed gene expression according to their functional roles and identity classifications. We found that the iNK cells exhibit lower expressions of NK activating and inflammatory features, including activating receptors, cytokines, chemokines, inflammatory and homing markers, and adhesion molecule gene groups, especially in non-activated and immature NK subsets, when compared with primary NK cells. Conversely, when examining these functional categories, mature and adaptive NK cell subsets exhibit fewer differences in gene expression (Fig. 5C). Collectively, this suggests that while there are differences in the gene expression of immature NK cell forms, the functional features become more closely aligned in the adaptive and mature forms, indicating a closer resemblance to their native counterparts upon maturation.

### The iNK cells exhibited potent cytotoxic activity against cholangiocarcinoma (CCA) and breast cancer (BCA) cell lines

**Fig. 6:**
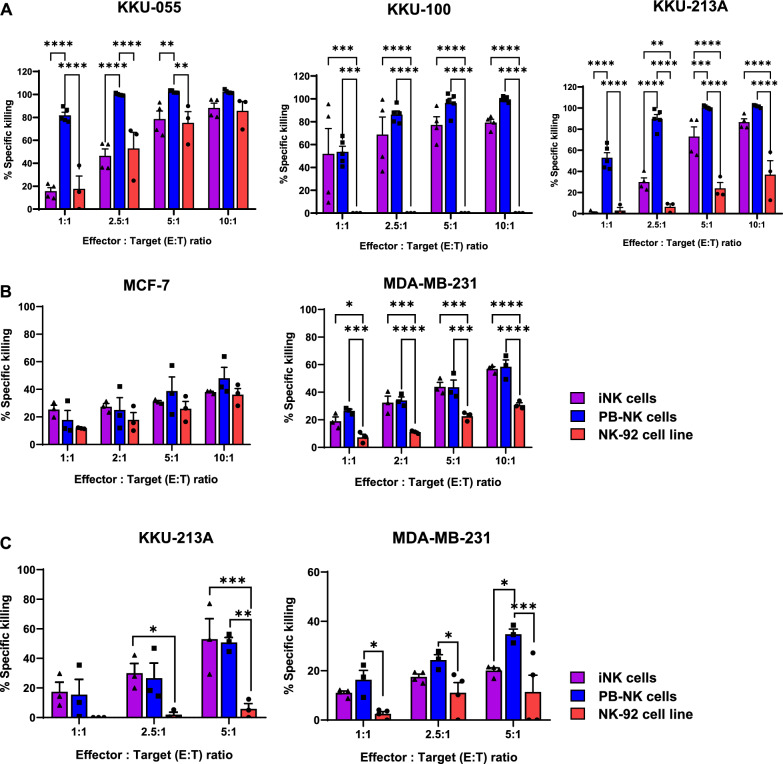
Analysis of effector functions of NK cells toward CCA and BCA cell lines. (**A**-**B**) Cytotoxic activity of NK cells against target cells after being co-cultured for 6 h. For cholangiocarcinoma, iNK cells (n = 4), PB-NK cells (n = 5 donors), and NK-92 cell line (n = 3). For BCA cell line iNK cells (n = 3), PB-NK cells (n = 3 donors), NK-92 cell line (n = 3). (**C**) Cytotoxic activity of NK cells against KKU-213A and MDA-MB-231 tumor spheroids after being co-cultured for 72 h. For KKU-213A; iNK cells n = 3, PB-NK cells n = 3 donors, NK-92 cells n = 3. For MDA-MB-231; iNK cells n = 4, PB-NK cells n = 3 donors, NK-92 cells n = 4. All data represent the mean ± SEM. Two-way ANOVA (Tukey’s multiple comparison test) was performed; ****p < 0.0001, *** p < 0.001, ** p < 0.01, * p < *0.05*

To evaluate the anti-tumor activity of NK cells, we co-cultured NK cells with CCA cell lines, including KKU-055, KKU-100, and KKU-213A, and BCA cell lines, including MCF-7 and MDA-MB-231, at various effector: target (E:T) ratios for 6 h (Fig. 6A, B). PB-NK cells showed the highest cytotoxic activity against the KKU-055, KKU-100, and KKU-213A cell lines. Even though the cytotoxic activity of the iNK cells was lower than that of the PB-NK cells, the iNK cells effectively eliminated the KKU-055, KKU-100, and KKU-213A cell lines in a dose-dependent manner. The iNK cells demonstrated significantly higher cytotoxicity toward the KKU-100 and KKU-213A cell lines compared to the NK-92 cell line at 2.5:1, 5:1, and 10:1 E:T ratios. Both the iNK cells and NK-92 cell line eliminated the KKU-055 cell line in a dose-dependent manner without significant differences in killing activity. Surprisingly, the cytotoxic activity of the NK-92 cell line against the KKU-213A cell line was only 36.9 ± 13.2% at a 10:1 E:T ratio, and the NK-92 cells could not eliminate the KKU-100 cell line even at the same ratio (Fig. 6A). All types of NK cells exhibited similar cytotoxic activity against the MCF-7 cell line. Regarding the MDA-MB-231 cell line, both iNK cells and PB-NK cells demonstrated similar effectiveness, both significantly greater than that of the NK-92 cell line (Fig. 6B). Furthermore, we examined IFN-γ and TNF-α secretion from all NK cells at 5:1 E:T ratio for CCA cell lines and IFN-γ secretion at 10:1 E:T ratio for BCA cell lines after 6 h of co-culture (Additional file 2: Fig. S8). The results showed that PB-NK cells elicited greater IFN-γ secretion compared to the iNK cells and the NK-92 cell line, though the differences were not statistically significant in both CCA and BCA cell lines. Variability in IFN-γ levels among PB-NK cells was observed, possibly due to differences among donors. PB-NK cells exhibited low levels of TNF-α secretion across all three target cells, while the iNK cells demonstrated the highest TNF-α secretion specifically against KKU-055 cells. However, no significant differences in TNF-α secretion were observed among the different types of NK cells.

Next, we evaluated the cytotoxic activity of the NK cells in a more physiologically relevant tumor model, using 3D tumor spheroids. The KKU-213A and MDA-MB-231 tumor spheroids were co-cultured with NK cells for 3 days at 1:1, 2.5:1, and 5:1 E:T ratios (Fig. 6C, D and Additional file 2: Fig. S9). In the presence of the iNK cells and PB-NK cells, we observed the PI-positive (red) tumor cells within the tumor spheroids, indicating that both types of cells could induce cytotoxicity. In contrast, the PI-positive tumor cells were not observed within tumor spheroids co-cultured with the NK-92 cell line. To quantify the anti-tumor activity, we evaluated the mean fluorescence intensity (MFI) of PI. The results showed that the iNK cells and PB-NK cells induced cell death in KKU-213A and MDA-MB-231 cells at comparable levels, and in a dose-dependent manner. However, PB-NK cells exhibited greater cytotoxicity against MDA-MB-231 cells compared to iNK cells at E:T ratio of 5:1. In contrast, the NK-92 cell line exhibited minimal cytotoxic activity, with only approximately 0.00 ± 0.00%, 1.92 ± 1.67%, and 5.84 ± 3.58% cell death in KKU-213A and 2.53 ± 0.90%, 11.09 ± 4.07%, and 11.36 ± 6.81% cell death in MDA-MB-231 at E:T ratios of 1:1, 2.5:1, and 5:1, respectively.

## Discussion

NK cells play a crucial role in the immune surveillance against tumor cells. Adoptive NK-cell immunotherapy has demonstrated safety and efficacy in numerous clinical studies [7]. However, the use of PB-NK cells in clinical trials faces some hurdles due to insufficient NK cell number and donor variability [25, 37]. Advancements in iPSC technology and the improved efficiency in generating iNK cells have made a translation of iPSC-derived NK cells for cancer therapy more feasible. Currently, the differentiation of iPSCs toward NK cells relies on the embryoid body (EB) and organoid-based methods [13, 38, 39]. However, these approaches have limitations due to the heterogeneity found in EB cultures, which can potentially impact the differentiation efficiency and reproducibility [21].

In this study, we developed a monolayer feeder-free method to generate NK cells from hiPSCs. Our protocol simplifies the differentiation process by reducing the technical hurdles associated with EB culture and decreasing medium consumption. During the initial HSPC differentiation process, we obtained high percentages of ME (KDR^+^ CD235a^−^ CD34^−^), HEPs (KDR^+^ CD235a^−^ CD34^+^ CD31^+^ CD144^+^), and HSPCs (CD34^+^ CD43^+^ CD45^±^). Thereafter, we extended the culture using an NK cell differentiation medium, which caused the cells to detach and become early hematopoietic cells expressing CD43 [40]. This detachment assisted in the purification of our desired cell type. Prolonged culture of these cells resulted in the expression of NK cell markers, such as CD56. Assessment of cellular markers during the differentiation process showed that the differentiated cells displayed generic markers resembling human NK cell development [29]. Nonetheless, NK cells generated using our protocol exhibited incomplete protein expression compared to their primary counterparts. For example, terminally differentiated iNK cells display high levels of CD56 and CD94, but low expression of CD57. In addition, we observed the expression of the progenitor marker, CD117, in some of our differentiations, suggesting that further investigations may be required to enhance maturation. Extended culture of differentiated iNK cells may allow for the acquisition of some mature or functional protein expression as has been demonstrated. Furthermore, we found that approximately 23—30% of the iNK cells expressed CD16. Such variations have been reported to be associated with hPSC lines rather than the differentiation protocol [38]. Screening iPSC lines for CD16 expression in iNK cells may be crucial in translational studies for cancer treatment.

Single-cell RNA sequencing of our differentiated iNK cells can help validate the cellular characteristics of our iNK cells, while also providing more comprehensive information on the varying states of NK cell maturation compared to primary NK cells. Our result demonstrated that the differentiated iNK cells yield several off-lineage cell types, such as eosinophils and myeloid cells. How such cells arise remains elusive and may be better understood with a more thorough study during the earlier stages of differentiation. A significant portion of the samples appears to be progenitor cells, which is consistent with our CD117 observation. Further optimizations and a deeper understanding of NK cell lineage specification could improve the differentiation efficiency.

Within the NK population itself, defined by generic markers for NK cells, we observed that the NK cell population appears to exhibit a broad and variable gene expression pattern. Sub-clustering of these cells allowed us to segregate these populations, which show a continuum of gene expression patterns, many of which are relevant in the context of maturation. Pseudotime analysis helped us delineate these maturation attributions, which we provide in our study as a resource for further investigations. Further exploration of these complex maturation gene patterns through their association with functional characteristics is essential, given the current uncertainty about which subtypes offer superior functionality.

The integration of single-cell data from primary NK cells with our iNK dataset provides a resource for comparing and examining differences between the two. Our study has demonstrated that while NK cells generated from iPSCs express key genes, they still lack many mature gene features in their primary counterparts [29]. While certain iNK subsets resemble specific primary NK subsets, such as adaptive and naïve states, it would be interesting to explore whether the difference we highlighted could enhance cell differentiation. Furthermore, it would be beneficial to determine whether these attributes could be tailored during differentiation to optimize NK cell maturation.

NK cell functions, including cytotoxic activity and cytokine production, are regulated by the integration of signals received from NK cell receptors. Typically, the imbalance of activating and inhibitory signals, provided by the interaction of their receptors and ligands on target cells, drives NK cell activity [41]. Upon differentiation, we achieved a high percentage of activating receptors on iNK cells such as NKp44, NKp46, and NKG2D. Engagement of these activating receptors with their ligands triggers the phosphorylation of the ITAM domain in the cytoplasmic tail, inducing NK cell activation [42]. Besides, the iNK cells express death ligands, including FasL and TRAIL, which can potentially induce target cell apoptosis upon binding with Fas and TRAIL receptors on target cells [43]. TIM3, an inhibitory receptor, was found to be highly expressed in the iNK cells. The expression of TIM3 could potentially inhibit the effector function of iNK cells through the interactions with Galectin-9 (Gal-9), CEACAM-1, HMGB1, and phosphatidylserine (PtdSer) [44]. Therefore, blocking or knocking out TIM-3 might be necessary to enhance the cytolytic activity of iNK cells, especially for TIM3^+^ tumor cells.

Cytotoxic activity against cancers is a crucial function of NK cells. In this study, we investigated the cytotoxic activity of iNK cells against three CCA cell lines and two BCA cell lines using both monolayer culture and 3D tumor spheroid culture systems, in comparison with PB-NK cells and the NK-92 cell line. In the monolayer culture system, our iNK cells exhibited lower cytotoxicity against all CCA cell lines compared to PB-NK cells. Nevertheless, in the spheroid culture system, which is more physiologically relevant to in vivo tumor models, iNK cells exhibited comparable cytotoxic activity against KKU-213A cells to PB-NK cells. Despite the immature phenotypes observed in our iNK cells, they exhibited superior cytotoxic activity compared to the NK-92 cell line, likely due to the higher levels of activating receptors present in the iNK cells. However, a limitation of this study is the lack of experiments conducted in animal models that exhibit more similar physiological characteristics of tumor biology to those in humans [45, 46]. Further research in such models is necessary to validate our findings.

## Conclusion

Collectively, our study established a simplified, cost-effective, and feeder-free monolayer protocol for generating iNK cells. This platform can create functional iNK cells expressing NK cell markers and a variety of activating and inhibitory receptors, potentially targeting various CCA and BCA cell lines. This iPSC-based approach provides an unlimited source of cells for generating NK cells for cancer treatment. In addition, when combined with gene editing technology such as the implementation of chimeric antigen receptors (CARs), overexpressing non-cleavable CD16, IL-15 superagonist and IL-15 receptor α (IL-15SA/IL-15RA), knocking out *CISH* gene or HLA-I molecule [17, 18, 47–51], or utilizing antibody therapy (52), this approach could provide off-the-shelf NK cell products that enhance the effectiveness of adoptive NK cell therapy.

## Supplementary Information

**Additional file 1**. Supplementary tables.

**Additional file 2**. Supplementary figures.

## Data Availability

Processed and raw single-cell RNA sequencing data have been deposited in the Gene Expression Omnibus (GEO) under accession code GSE270690. GRCh38 human reference genome was used. Other supporting data are available from the corresponding author upon reasonable request.

## Abbreviations

ACT: Adoptive cell therapy
ADCC: Antibody-dependent cellular cytotoxicity
AML: Acute myeloid leukemia
ANOVA: Analysis of variances
BCA: Breast cancer
CAR: Chimeric antigen receptor
CCA: Cholangiocarcinoma
CD: Cluster of differentiation
CFSE: Carboxyfluorescein succinimidyl ester
EB: Embryoid body
EHT: Endothelial-to-hematopoietic transition
ELISA: Enzyme-linked immunosorbent assay
ESC: Embryonic stem cell
FACS: Fluorescent activated cell sorting
FBS: Fetal bovine serum
FcR: Fc receptor
FGF2: Fibroblast growth factor 2
Flt3L: Fms-related tyrosine kinase 3 ligand
GvHD: Graft-versus-host disease
HEP: Hematoendothelial progenitor
HLA: Human leukocyte antigen
HSPC: Hematopoietic stem/progenitor cell
IFN: Interferon
IL: Interleukin
iNK: Induced pluripotent stem cell-derived natural killer cells
iPSC: Induced pluripotent stem cell
KIR: Killer-cell immunoglobulin-like receptor
LAG-3: Lymphocyte activation gene 3
ME: Mesoderm
MFI: Mean fluorescence intensity
NCAM: Neural cell adhesion molecule
NCR: Natural cytotoxicity receptor
NGS: Next-generation sequencing
NK: Natural killer cell
PB: Peripheral blood
PBMC: Peripheral blood mononuclear cell
PBS: Phosphate buffer saline
PD-1: Programmed death 1
qPCR: Quantitative polymerase chain reaction
SCF: Stem cell factor
TGF: Transforming growth factor
TIGIT: T cell immunoreceptor with immunoglobulin and Immunoreceptor tyrosine-based inhibitory motif domain
TIM-3: T-cell immunoglobulin and mucin-domain containing 3
TNF: Tumor necrosis factor
tSNE: t-distributed stochastic neighbour embedding
UCB: Umbilical cord blood
VEGF: Vascular endothelial growth factor
