# Supplementary material for: Phenotypic and transcriptomic profiling of induced pluripotent stem cell (iPSC)-derived NK cells and their cytotoxicity against cancers
Source: Stem Cell Res Ther. 2024 Nov 13;15:418. doi: 10.1186/s13287-024-04029-z (PMC11559060; doi:10.1186/s13287-024-04029-z)
Supplement: Supplementary file 1 — Additional file 1. Supplementary tables. [file 13287_2024_4029_MOESM1_ESM.docx]

**Additional file 1: Supplementary tables**

**Phenotypic and Transcriptomic Profiling of Induced Pluripotent Stem Cell (iPSC)-Derived NK Cells and Their Cytotoxicity against Cancers**

Nontaphat Thongsin^1,2^, Siriwal Suwanpitak^1^, Punn Augsornworawat^2^, Jakkrapatra Srisantitham^1,2^, Kritayaporn Saiprayong^1^, Piroon Jenjaroenpun^3^, Methichit Wattanapanitch^1,*^

^1^Siriraj Center for Regenerative Medicine, Research Department, Faculty of Medicine Siriraj Hospital, Mahidol University, Thailand

^2^Department of Immunology, Faculty of Medicine Siriraj Hospital, Mahidol University, Thailand

^3^Division of Bioinformatics and Data Management for Research, Research Department, Faculty of Medicine Siriraj Hospital, Mahidol University, Thailand

*Correspondence: methichit.wat@mahidol.ac.th

**Table S1**. **Primer sequence for RT-qPCR.**

| **Target** | **Primer set** | **Reference** |
| --- | --- | --- |
| *OCT4* | Forward: TCGAGAACCGAGTGAGAGG  Reverse: GAACCACACTCGGACCACA | (52) |
| *NANOG* | Forward: AGATGCCTCACACGGAGACT  Reverse: GGACTGGTGGAAGAATCAGG | (52) |
| *Brachyury* | Forward: GCTGTGACAGGTACCCAACC  Reverse: CATGCAGGTGAGTTGTCAGAA | (52) |
| *KDR* | Forward: TGAGCAAAGGGTGGAGGTGACT  Reverse: CTTGCACAAAGTGACACGTTGAG | (52) |
| *GAPDH* | Forward: GTCAACGGATTTGGTCGTATTG  Reverse: CATGGGTGGAATCATATTGGAA | (52) |

**Table S2. Antibodies used for flow cytometric analysis.**

| **Antibody** | **Conjugated dye** | **Dilution** | **Catalog number** | **Manufacturer** |
| --- | --- | --- | --- | --- |
| Anti-human CD309 (KDR) | APC | 1:50 | 359916 | BioLegend |
| Anti-human CD235a | PE | 1:50 | 349106 | BioLegend |
| Anti-human CD34 | Alexa Fluor 700 | 1:50 | 343526 | BioLegend |
| Anti-human CD43 | PE/Cy7 | 1:50 | 343208 | BioLegend |
| Anti-human CD45 | PerCP | 1:50 | 368506 | BioLegend |
| Anti-human CD31 | Pacific Blue | 1:50 | 130-106-503 | BioLegend |
| Anti-human CD144 | FITC | 1:50 | 130­100­742 | Miltenyi Biotec |
| Anti-human CD3 | FITC | 1:50 | 300406 | BioLegend |
| Anti-human CD56 | PE/Cy7 | 1:50 | 362509 | BioLegend |
| Anti-human CD94 | APC | 1:50 | 305508 | BioLegend |
| Anti-human CD7 | Alexa Fluor 700 | 1:50 | 343125 | BioLegend |
| Anti-human CD117 | Brilliant Violet 510 | 1:50 | 313220 | BioLegend |
| Anti-human CD16 | Brilliant Violet 650 | 1:50 | 302041 | BioLegend |
| Anti-human CD107a | APC/Cy7 | 1:50 | 328630 | BioLegend |
| Anti-human CD57 | PE | 1:50 | 393307 | BioLegend |
| Anti-human CD158 (KIRs) | FITC | 1:50 | 339503 | BioLegend |
| Anti-human NKp44 | APC | 1:50 | 325109 | BioLegend |
| Anti-human NKp46 | PE | 1:50 | 331907 | BioLegend |
| Anti-human NKG2A | PE | 1:50 | FAB1059P-025 | R&D Systems |
| Anti-human NKG2C | Alexa fluor 488 | 1:50 | FAB138G-025 | R&D Systems |
| Anti-human NKG2D | APC/Cy7 | 1:50 | 320823 | BioLegend |
| Anti-human TRAIL | APC | 1:50 | 308209 | BioLegend |
| Anti-human FasL | Brilliant Violet 421 | 1:50 | 306411 | BioLegend |
| Mouse IgG1, κ isotype control | APC | 1:50 | 400120 | BioLegend |
| Mouse IgG1, κ isotype control | PE | 1:50 | 400112 | BioLegend |
| Mouse IgG1, κ isotype control | Alexa Fluor 700 | 1:50 | 400143 | BioLegend |
| Mouse IgG1, κ isotype control | PE/Cy7 | 1:50 | 400125 | BioLegend |
| Mouse IgG1, κ isotype control | PerCP | 1:50 | 400147 | BioLegend |
| Mouse IgG2b, κ isotype control | Pacific Blue | 1:50 | 400331 | BioLegend |
| REA Control | FITC | 1:50 | 130-104-610 | Miltenyi Biotec |
| Mouse IgG1, κ isotype control | FITC | 1:50 | 400107 | BioLegend |
| Mouse IgG2a, κ isotype control | Alexa Fluor 700 | 1:50 | 400247 | BioLegend |
| Mouse IgG1, κ isotype control | Brilliant Violet 510 | 1:50 | 400171 | BioLegend |
| Mouse IgG1, κ isotype control | Brilliant Violet 650 | 1:50 | 400163 | BioLegend |
| Mouse IgG1, κ isotype control | APC/Cy7 | 1:50 | 400127 | BioLegend |
| Mouse IgG2b, κ isotype control | FITC | 1:50 | 401205 | BioLegend |
| Mouse IgG1, κ isotype control | Alexa fluor 488 | 1:50 | IC002G | R&D Systems |
| Mouse IgG1, κ isotype control | Brilliant Violet 421 | 1:50 | 400157 | BioLegend |
| Zombie Violet™ Viability Kit | Pacific Blue | 1:500 | 423113 | BioLegend |
| Zombie Aqua™ Viability Kit | Brilliant Violet 510 | 1:500 | 423101 | BioLegend |
