# Supplementary material for: Phenotypic and transcriptomic profiling of induced pluripotent stem cell (iPSC)-derived NK cells and their cytotoxicity against cancers
Source: Stem Cell Res Ther. 2024 Nov 13;15:418. doi: 10.1186/s13287-024-04029-z (PMC11559060; doi:10.1186/s13287-024-04029-z)
Supplement: Supplementary file 2 — Additional file 2. Supplementary figures. [file 13287_2024_4029_MOESM2_ESM.docx]

**Additional file 1: Supplementary figures**

**Phenotypic and Transcriptomic Profiling of Induced Pluripotent Stem Cell (iPSC)-Derived NK Cells and Their Cytotoxicity against Cancers**

Nontaphat Thongsin^1,2^, Siriwal Suwanpitak^1^, Punn Augsornworawat^2^, Jakkrapatra Srisantitham^1,2^, Kritayaporn Saiprayong^1^, Piroon Jenjaroenpun^3^, Methichit Wattanapanitch^1,*^

^1^Siriraj Center for Regenerative Medicine, Research Department, Faculty of Medicine Siriraj Hospital, Mahidol University, Thailand

^2^Department of Immunology, Faculty of Medicine Siriraj Hospital, Mahidol University, Thailand

^3^Division of Bioinformatics and Data Management for Research, Research Department, Faculty of Medicine Siriraj Hospital, Mahidol University, Thailand

*Correspondence: methichit.wat@mahidol.ac.th

**
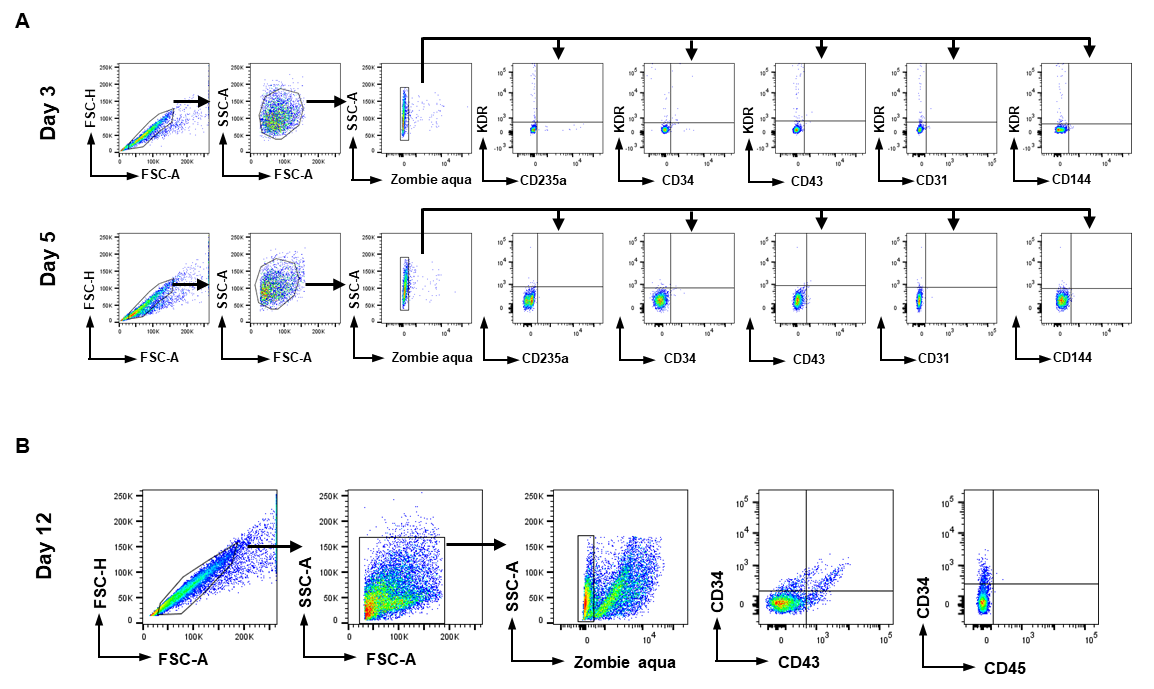
**

**Figure S1. Gating strategy for identifying ME, HEP, and HSPCs.** Dot plot analysis represents the main gating strategy for identifying (A) ME and HEP on days 3 and 5 and (B) HSPCs on day 12 of differentiation.

**
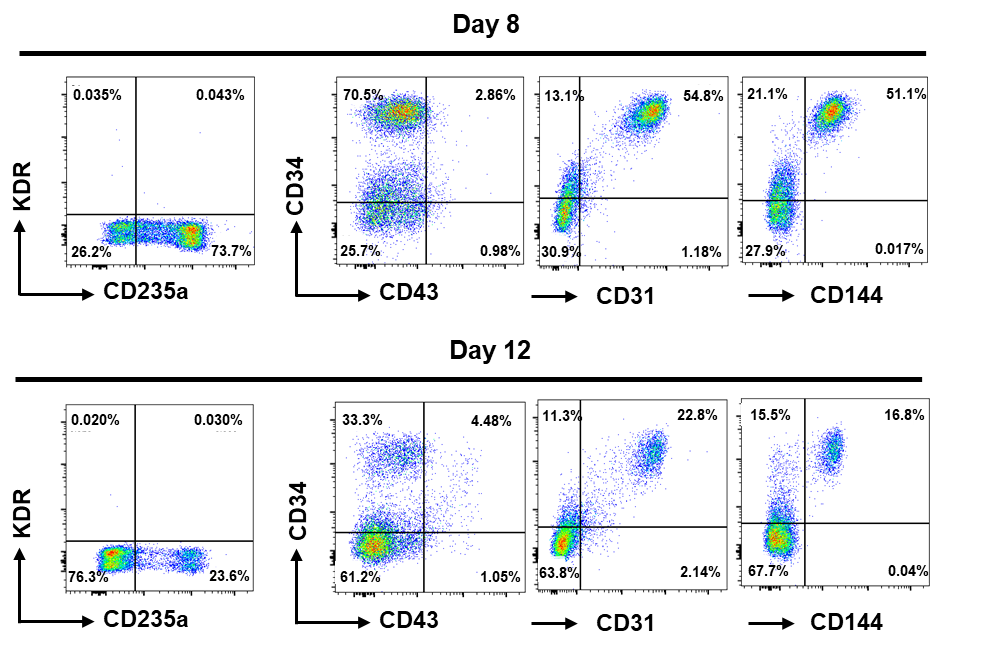
**

**Figure S2. Phenotypic analysis of non-floating cells.** Flow cytometric analysis shows the phenotye of non-floating cells on days 8 and 12 of differentiation. The positive populations in the flow cytometric data were gated using the corresponding isotype controls.

**
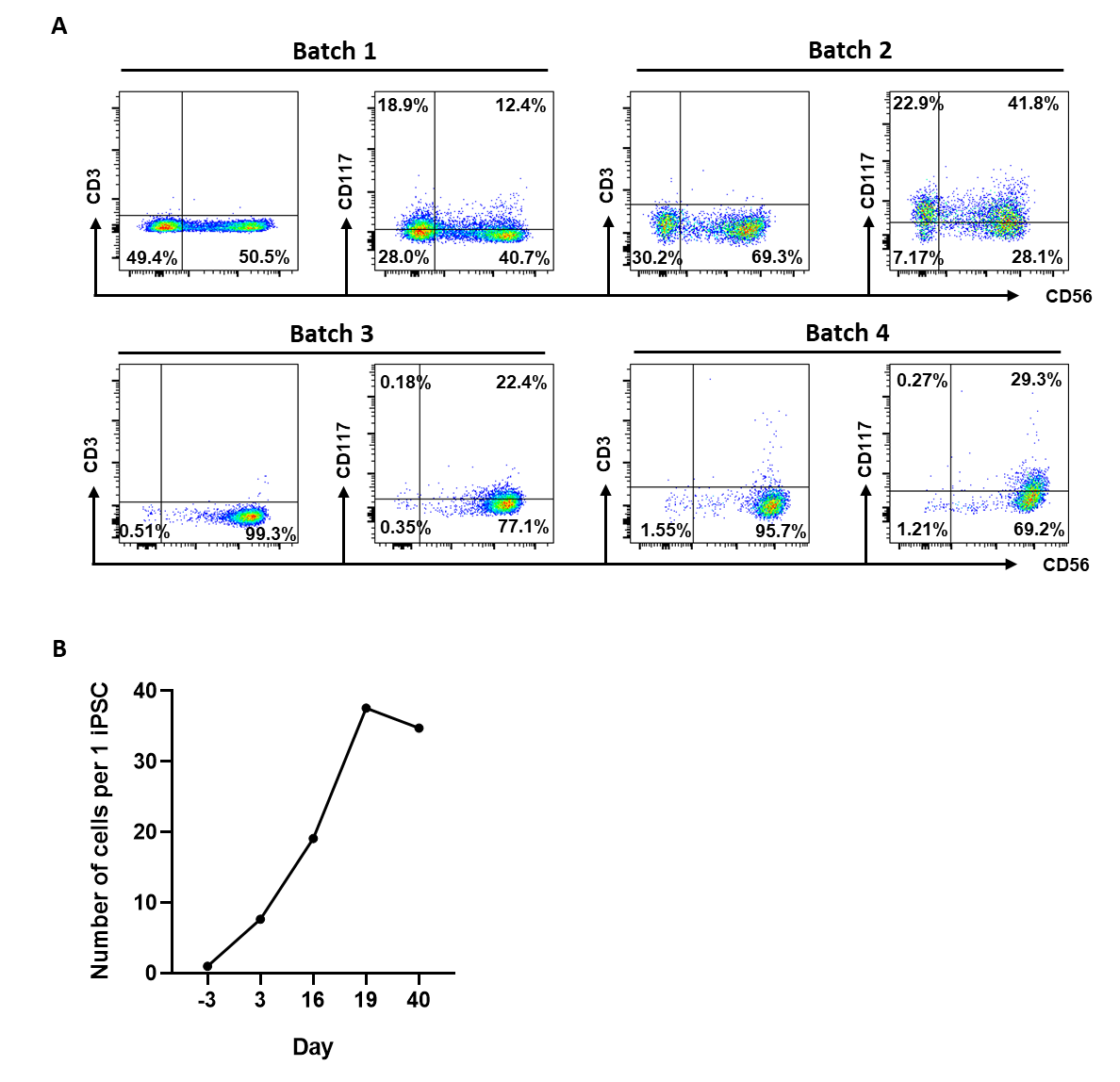
**

**Figure S3. Phenotypic analysis of immature iNK cells and the cell number.** (A) Flow cytometric analysis shows the diversity of the iNK cells across various differentiation batches on day 40. The positive populations in the flow cytometric data were gated using the corresponding isotype controls. (B) The number of cells generated on each day of differentiation from a single iPSC.

**
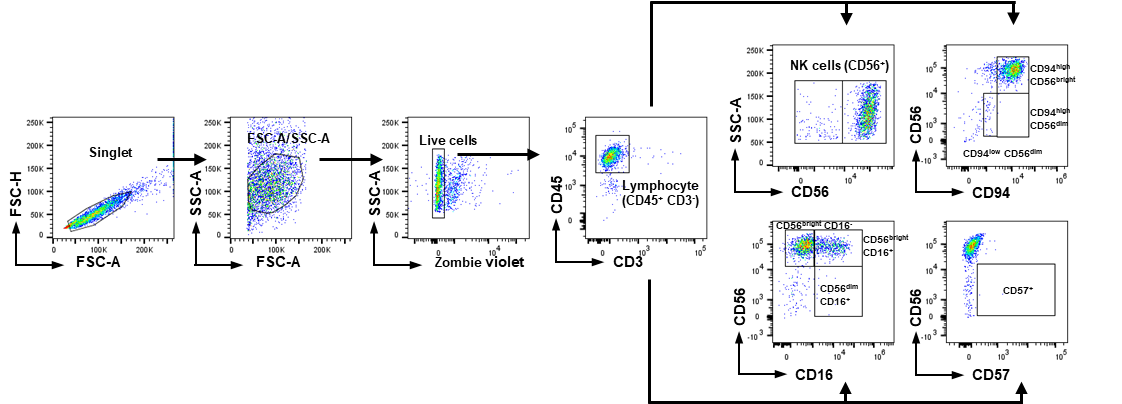
**

**Figure S4. Gating strategy for identifying NK cell subpopulations.** Dot plot analysis represents the main gating strategy for NK cells. NK cells were identified as CD45^+^ CD3^-^ CD56^+^ cells. The positive subsets within the flow cytometric data were gated using the corresponding isotype controls.


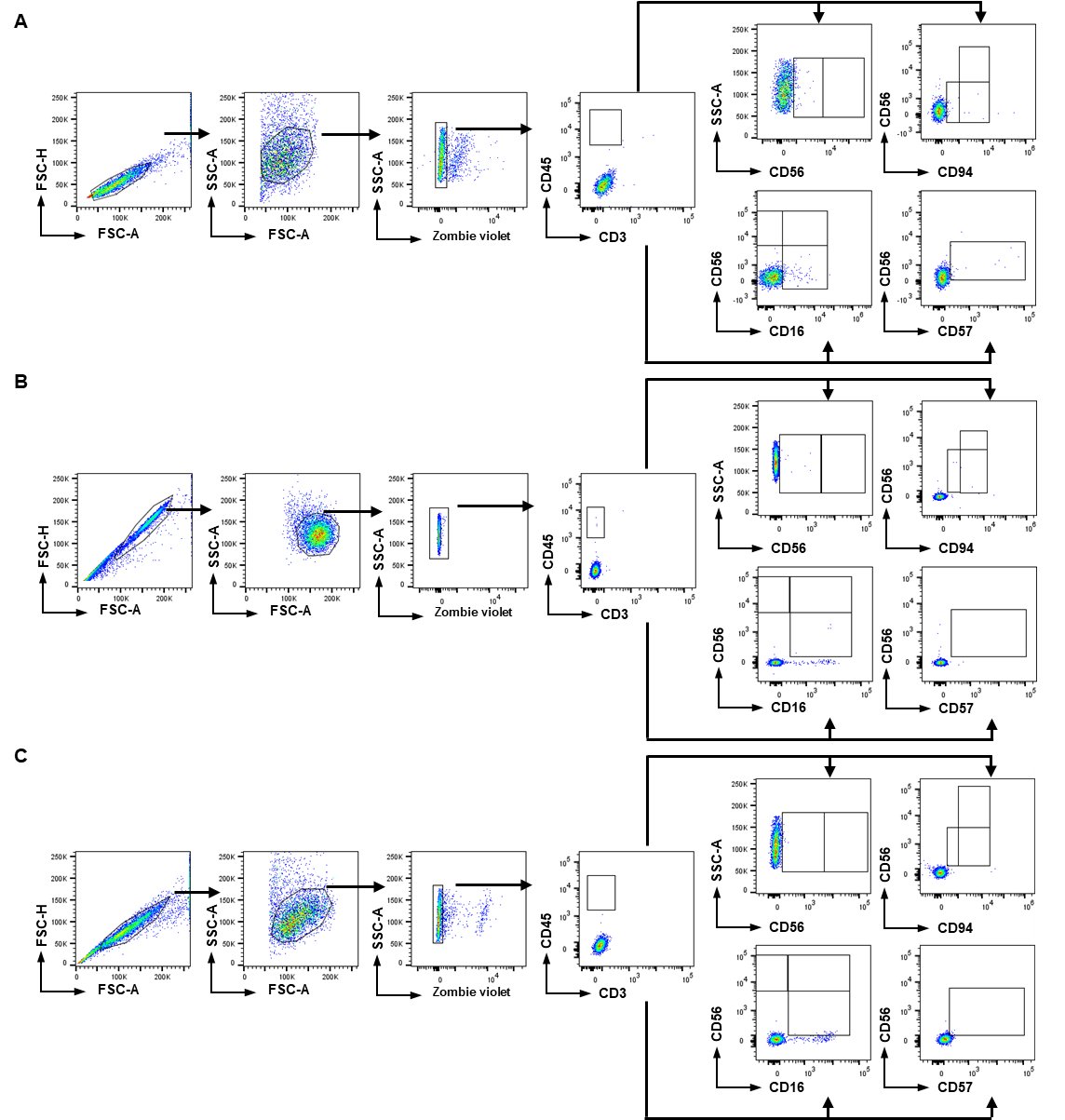


**Figure S5. Gating strategy for identifying NK cell subpopulations.** Dot plot analysis represents the isotype control for (**A**) iNK cells, (**B**) PB-NK cells, and (**C**) NK-92 cell line.

**
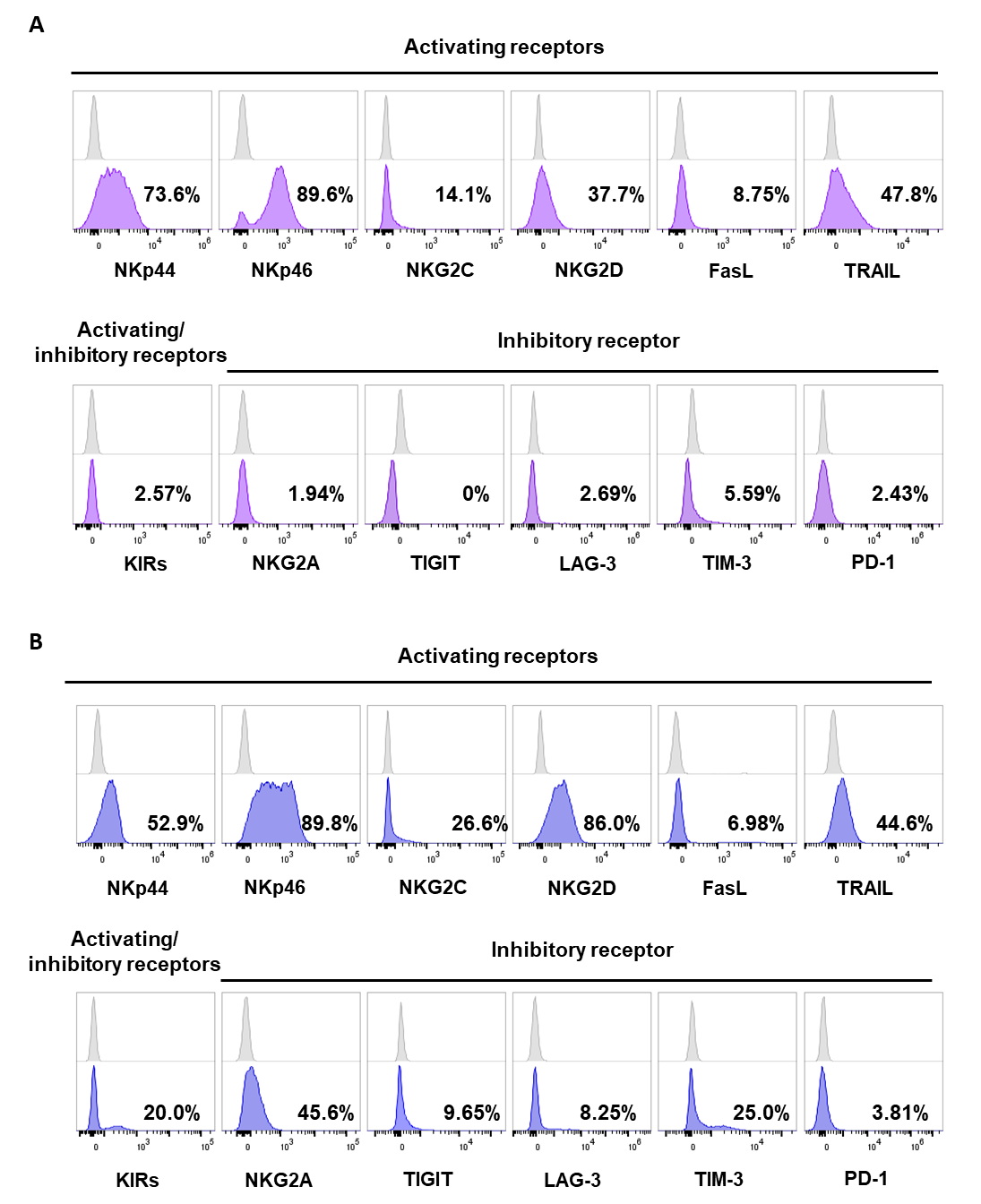
**

**Figure S6. Expression of NK cell activating and inhibitory receptors on (A) iNK cells and (B) PB-NK cells.** The positive populations in the flow cytometry data were gated using the corresponding isotype controls. The plots are representative of one experiment out of a total of 2 independent experiments. The anti-KIRs monoclonal antibody is an antibody cocktail targeting an inhibitory receptor KIR2DL1, and the activating receptors KIR2DS2, KIR2DS3, KIR2DS5.

**
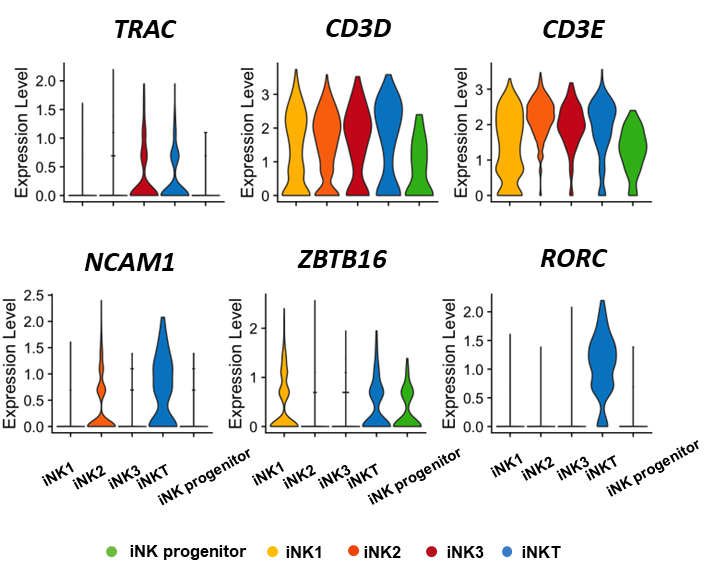
**

**Figure S7. Violin plots represent gene expression associated with NKT cells.**

**
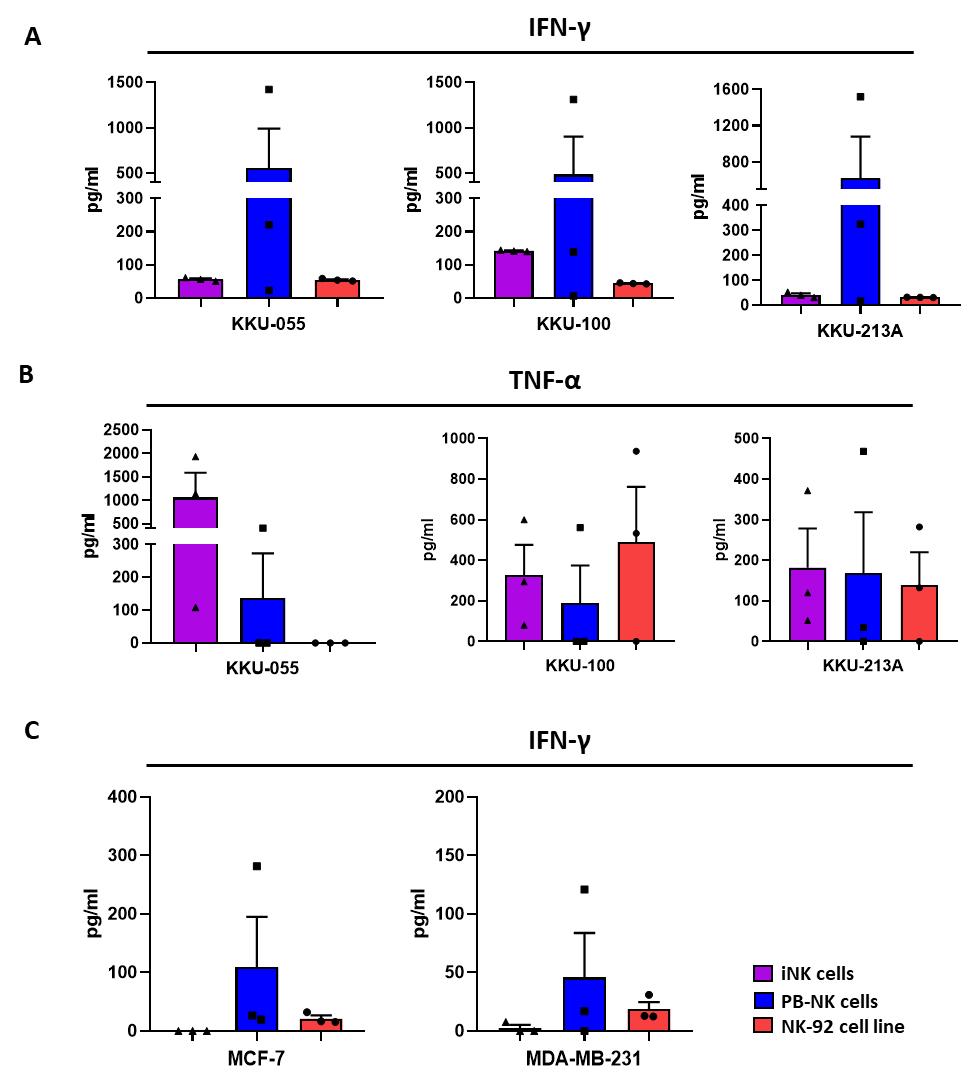
**

**Figure S8. Cytokine secretion of NK cells after co-culturing with CCA and BCA cell lines, as analyzed by ELISA.** The amount of IFN-γ and TNF-α were determined after 6 h of co-culture. For iNK cells, data were collected from 3 independent experiments, PB-NK cells from 3 different donors, and for the NK-92 cell line, n = 3. All graphs represent the mean values ± SEM. Statistical analysis was performed using one-way ANOVA with Tukey’s multiple comparison test.


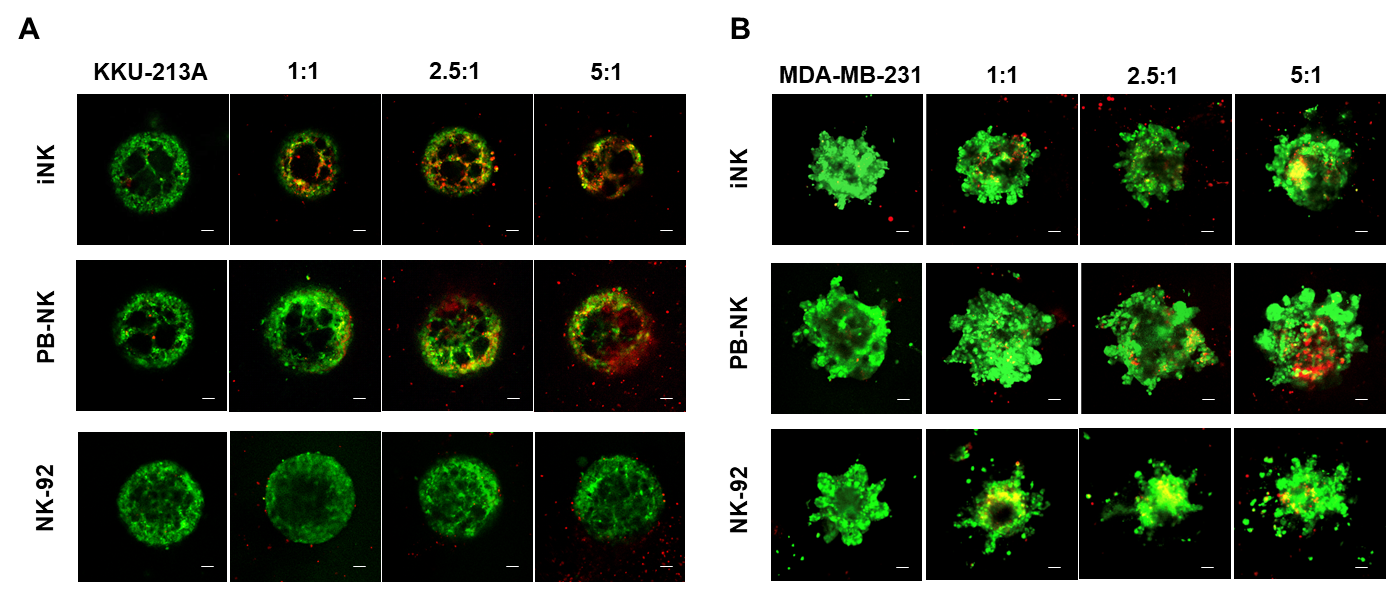


**Figure S9. Cytotoxic activity of NK cells against KKU-213A and MDA-MB-231 tumor spheroids after being co-cultured for 72 h.** Representative fluorescent images show tumor cells in green color (CFSE) and dead cells in red color (PI). Scale bar = 100 μm. The images are representative of one experiment among a total of 3 or 4 experiments conducted (n = 3 or 4).
